# Supplementary material for: Pollen sterols are associated with phylogeny and environment but not with pollinator guilds
Source: New Phytol. 2021 Feb 15;230(3):1169–84. doi: 10.1111/nph.17227 (PMC8653887; doi:10.1111/nph.17227)
Supplement: Supplementary file 1 — Fig. S1 GC‐MS spectra of the 25 phytosterols identified in our study (after Tri‐sil derivatisation, extraction details see the Materials and Methods section). Fig. S2 2D‐NMDS plot: pollen sterol profile similarities between species of different plant families. Fig. S3 24‐methylenecholesterol content of plants without pollen as bee or with pollen as reward for bees. Fig. S4 Total sterol content of plants without pollen as bee reward or with pollen as reward for bees. [file NPH-230-1169-s002.pdf]

New Phytologist

New Phytologist **Supporting Information**

Article title:

Pollen sterols are associated with phylogeny and environment but not with pollinator guilds

Authors:

Pengjuan Zu, Hauke Koch, Orlando Schwery, Samuel Pironon, Charlotte Phillips, Ian Ondo, Iain W. Farrell, W. David Nes, Elynor Moore, Geraldine A. Wright, Dudley I. Farman, Philip C. Stevenson

Article acceptance date: 14 January 2021

The following Supporting Information is available for this article:

Supplementary figures:

Fig. S1. GC-MS spectra of the 25 phytosterols identified in our study (after Tri-sil derivatisation, extraction details see Materials and methods section). RRT_c stands for the relative retention time to cholesterol (calculated by absolute retention time of the specific compound devided by absolute retention time of cholesterol). *Note that this figure includes multiple pages are attached at the end of the document.*

Fig. S2. 2D-NMDS (non-metric multidimensional scaling) plot of pollen sterol profile similarities (Bray-Curtis) between plant species with major plant families highlighted with different symbols (see legend). Distances between points correspond to sterol profile dissimilarities.

Fig. S3. 24-methylenecholesterol content (µg/mg pollen) of plants without pollen as bee reward (no bee pollination/collection of pollen by bees; n = 22), or with pollen as reward for bees (based on evidence of bee pollination and pollen collection by bees; n = 54). Black line: median; box: inter-quartile range (IQR); whiskers extend to highest value within 1.5 * IQR from upper quartile, dots: outliers beyond 1.5 * IQR from upper quartile.

Fig. S4. Total sterol content (µg/mg pollen) of plants without pollen as bee reward (n = 22), or with pollen as reward for bees (n = 54). Indentations represent 95% confidence intervals. Black line: median; box: inter-quartile range (IQR); whiskers extend to highest/lowest value within 1.5 * IQR from upper/lower quartile, dots: outliers beyond 1.5 * IQR from upper quartile.

Supporting information tables (see separate file):

Table S1. Data table (plant species, scores for different environmental variables/principal components, pollination guilds, sterol composition including both relative & absolute amounts.

Table S2. Scientific name and family for all sampled species, along with suggested OTL synonyms (which were subsequently used) and taxon IDs; species excluded from the phylogeny are highlighted in grey; reason for exclusion due to issues in the data and/or the OTL taxonomy are indicated.

Table S3. Variable contributions to axes of PCA of 13 environmental variables.

Table S4. Results of linear model tests for phylogenetic independent contrasts (PICs) of total sterol amount/diversity against PICs of environmental variables and niche volume.

Data availability

All the source data used for this publication can be found in Supporting Information tables. Related codes for analyses can be found at: http://doi.org/10.5281/zenodo.4448216.

Fig. S1 *(See pages after Fig. S4, from page 6)*

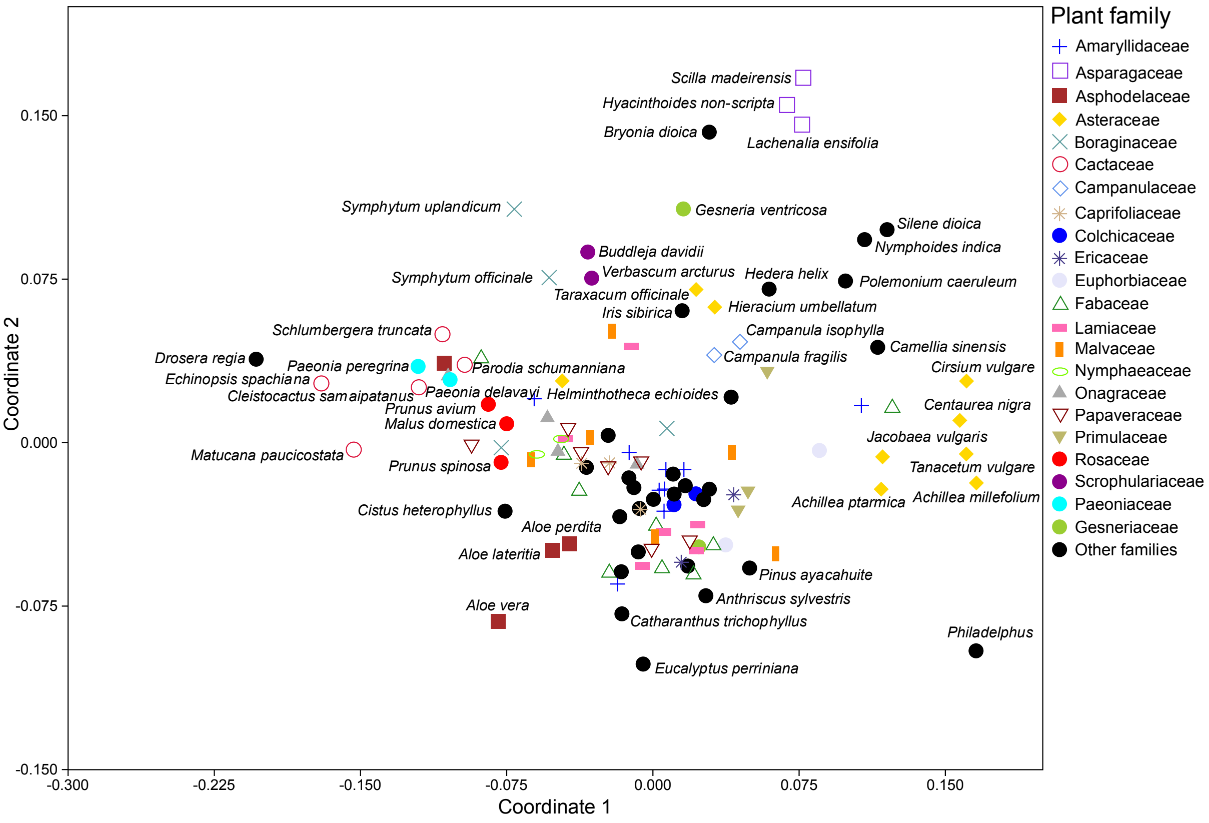

Fig. S2. 2D-NMDS (non-metric multidimensional scaling) plot of pollen sterol profile similarities (Bray-Curtis) between plant species with major plant families highlighted with different symbols (see legend). Distances between points correspond to sterol profile dissimilarities.

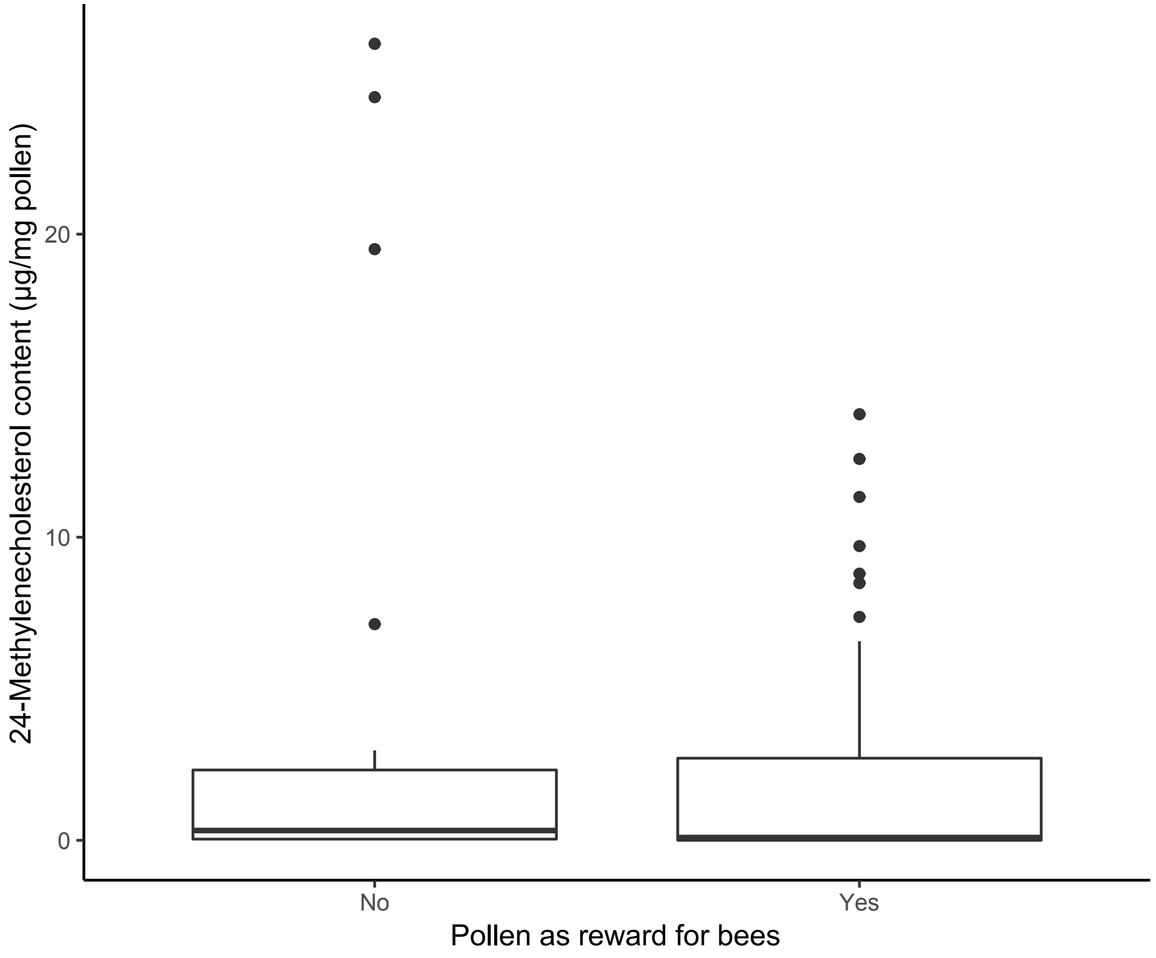

Fig. S3. 24-methylenecholesterol content (µg/mg pollen) of plants without pollen as bee reward (no bee pollination/collection of pollen by bees; n = 22), or with pollen as reward for bees (based on evidence of bee pollination and pollen collection by bees; n = 54). Black line: median; box: inter-quartile range (IQR); whiskers extend highest value within 1.5 * IQR from upper quartile, dots: outliers beyond 1.5 * IQR from upper quartile.

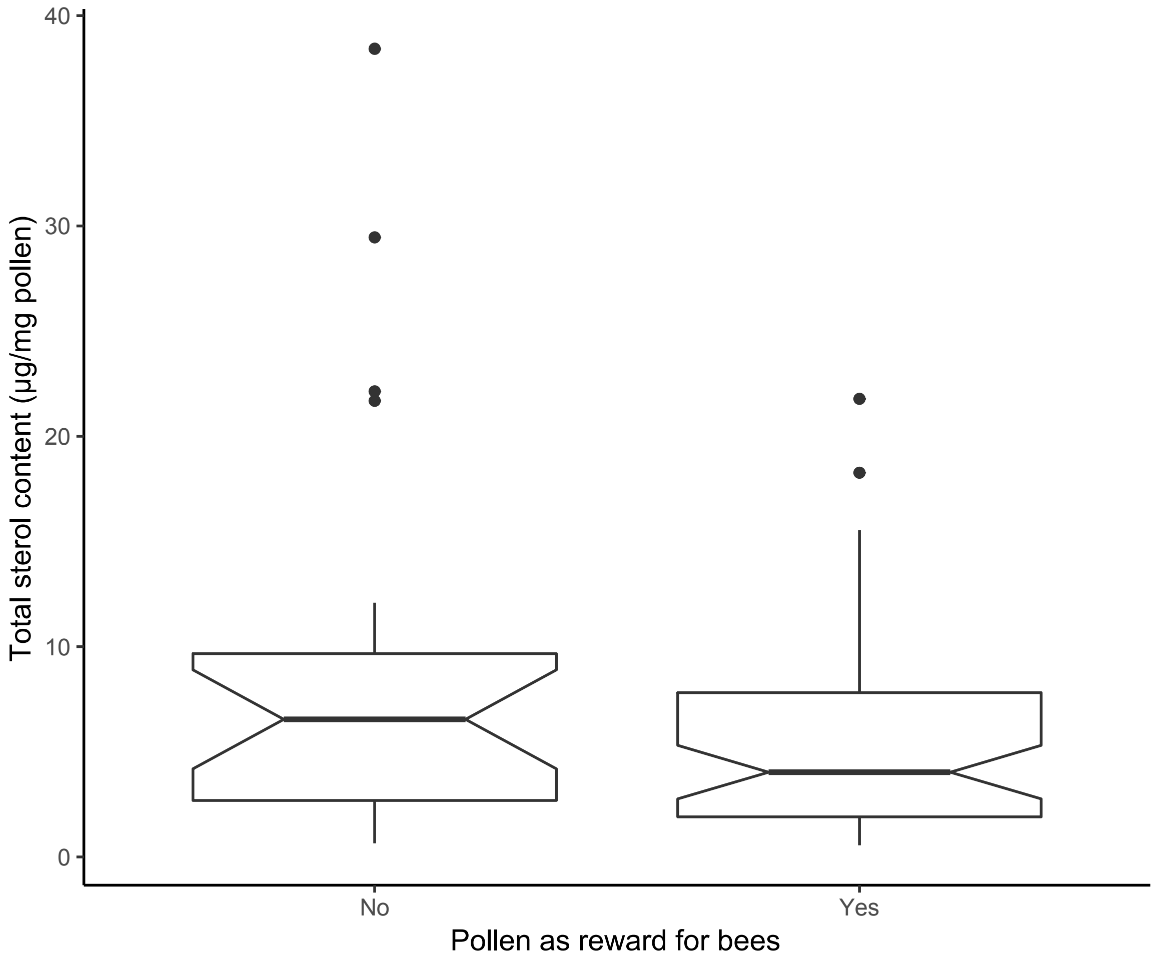

Fig. S4. Total sterol content (µg/mg pollen) of plants without pollen as bee reward (n = 22), or with pollen as reward for bees (n = 54). Indentations represent 95% confidence intervals. Black line: median; box: inter-quartile range (IQR); whiskers extend highest/lowest value within 1.5 * IQR from upper/lower quartile, dots: outliers beyond 1.5 * IQR from upper quartile.

Fig. S1. GC-MS spectra of the 25 phytosterols identified in our study (after Tri-sil derivatisation, extraction details see Materials and methods section). RRT_c stands for the relative retention time to cholesterol (calculated by absolute retention time of the specific compound devided by absolute retention time of cholesterol).

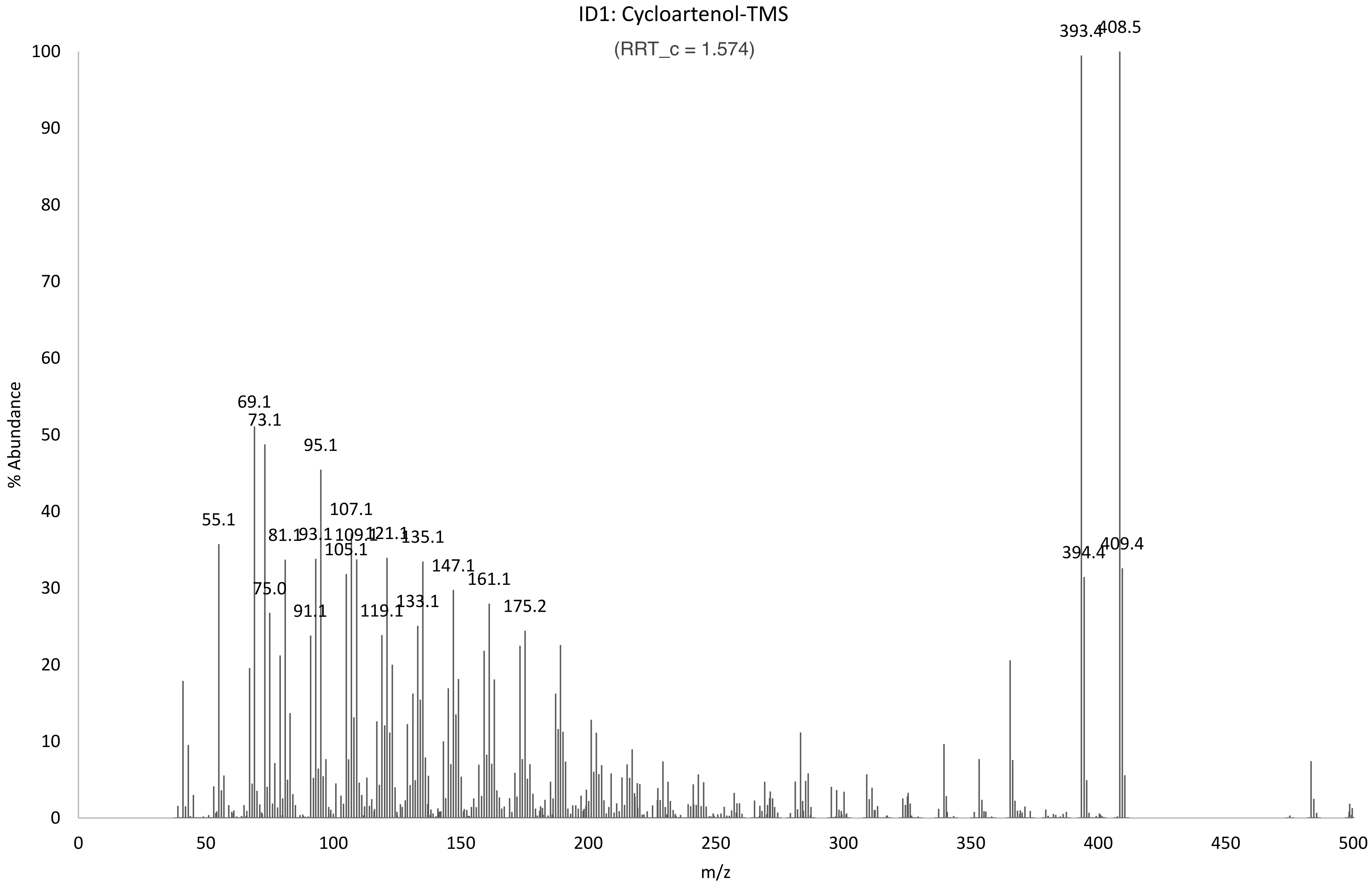

ID1: Cycloartenol-TMS
(RRT_c = 1.574)
% Abundance
m/z
0
10
20
30
40
50
60
70
80
90
100
0
50
100
150
200
250
300
350
400
450
500
55.1
69.1
73.1
75.0
81.1
91.1
93.1
95.1
105.1
107.1
109.1
119.1
121.1
133.1
135.1
147.1
161.1
175.2
393.4
394.4
408.5
409.4

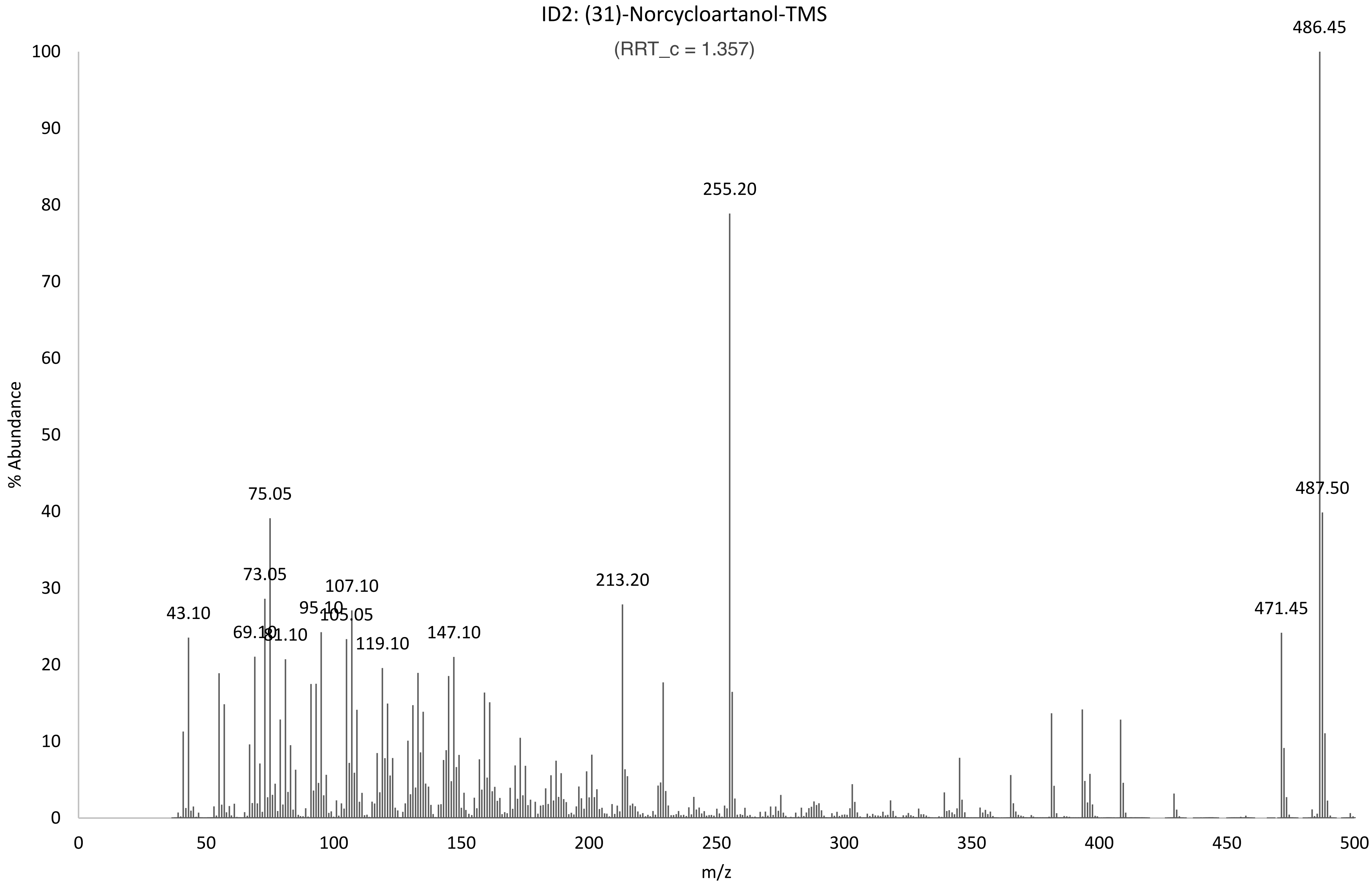
ID2: (31)-Norcycloartanol-TMS
(RRT_c = 1.357)
% Abundance
m/z
486.45
255.20
487.50
75.05
73.05
107.10
213.20
43.10
95.10
105.05
471.45
69.10
81.10
147.10
119.10
0
10
20
30
40
50
60
70
80
90
100
0
50
100
150
200
250
300
350
400
450
500

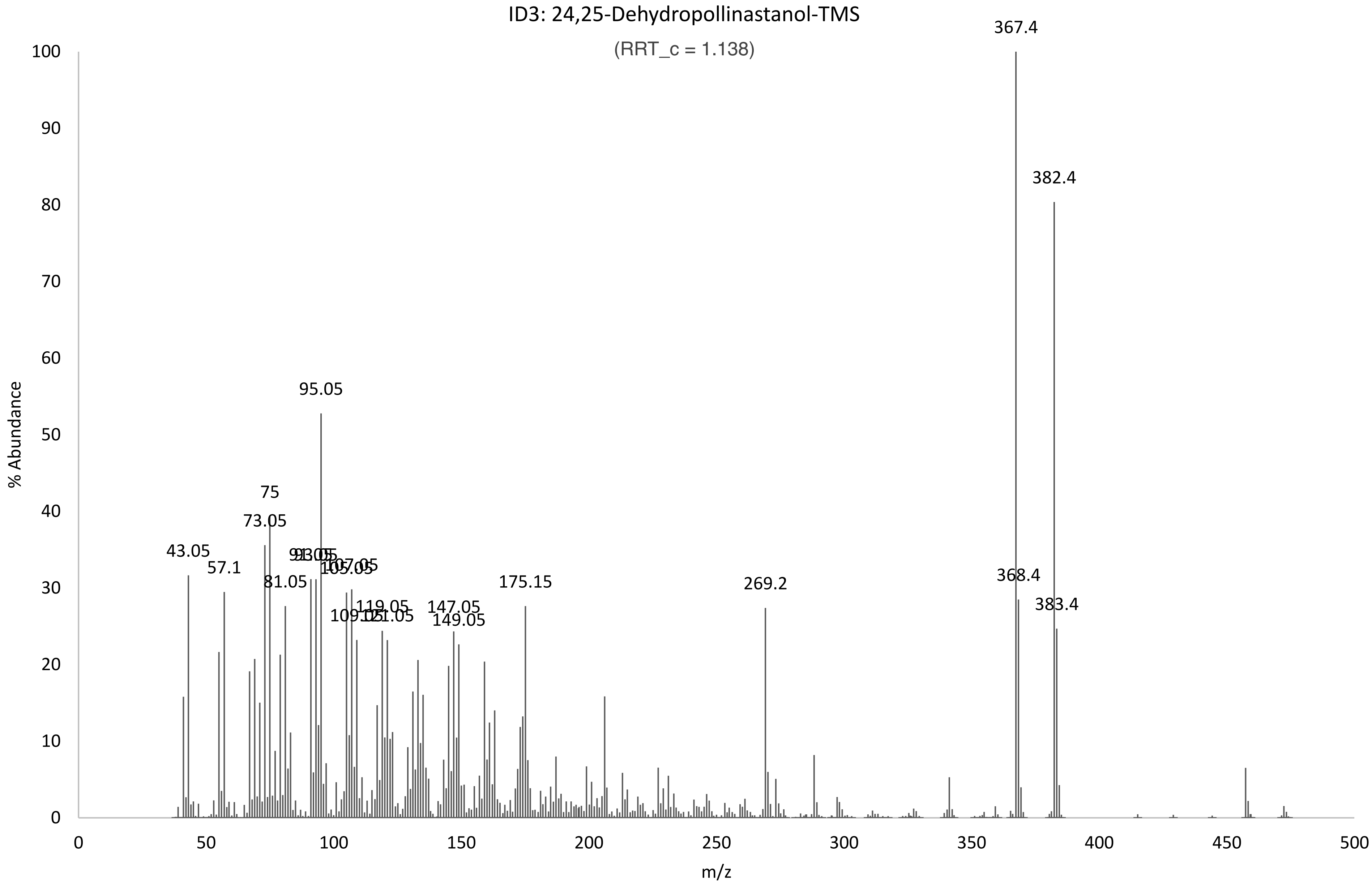
ID3: 24,25-Dehydropollinastanol-TMS
(RRT_c = 1.138)
% Abundance
m/z
367.4
382.4
95.05
75
73.05
43.05
57.1
81.05
91.05
93.05
105.05
107.05
109.05
119.05
121.05
147.05
149.05
175.15
269.2
368.4
383.4

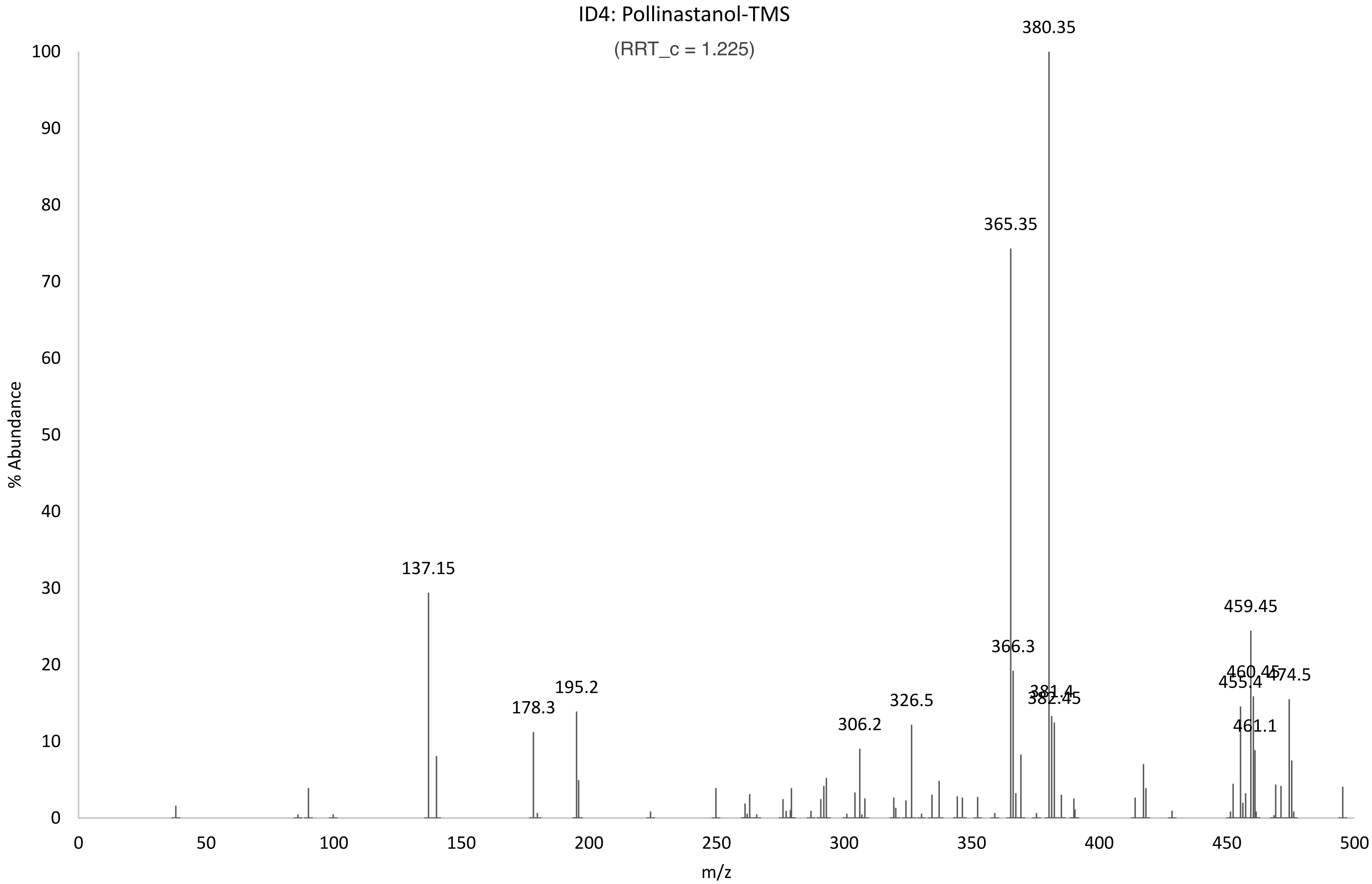
ID4: Pollinastanol-TMS
(RRT_c = 1.225)
% Abundance
m/z
380.35
365.35
137.15
459.45
366.3
195.2
178.3
381.4
382.45
326.5
306.2
460.45
455.4
474.5
461.1
0
10
20
30
40
50
60
70
80
90
100
0
50
100
150
200
250
300
350
400
450
500

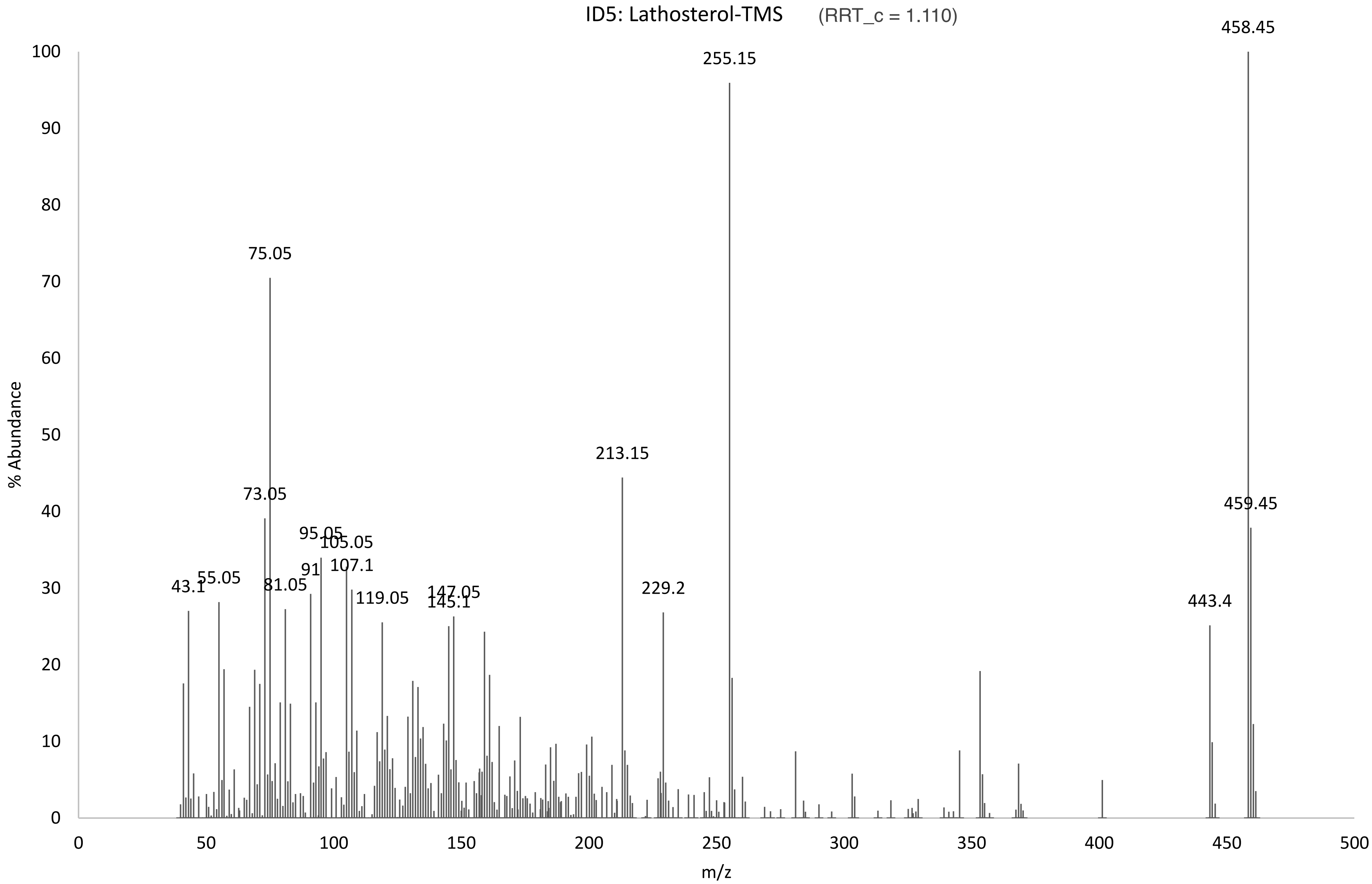
ID5: Lathosterol-TMS
(RRT_c = 1.110)
% Abundance
m/z
0
10
20
30
40
50
60
70
80
90
100
0
50
100
150
200
250
300
350
400
450
500
458.45
255.15
75.05
213.15
73.05
459.45
95.05
105.05
91
107.1
43.1
55.05
81.05
119.05
147.05
145.1
229.2
443.4

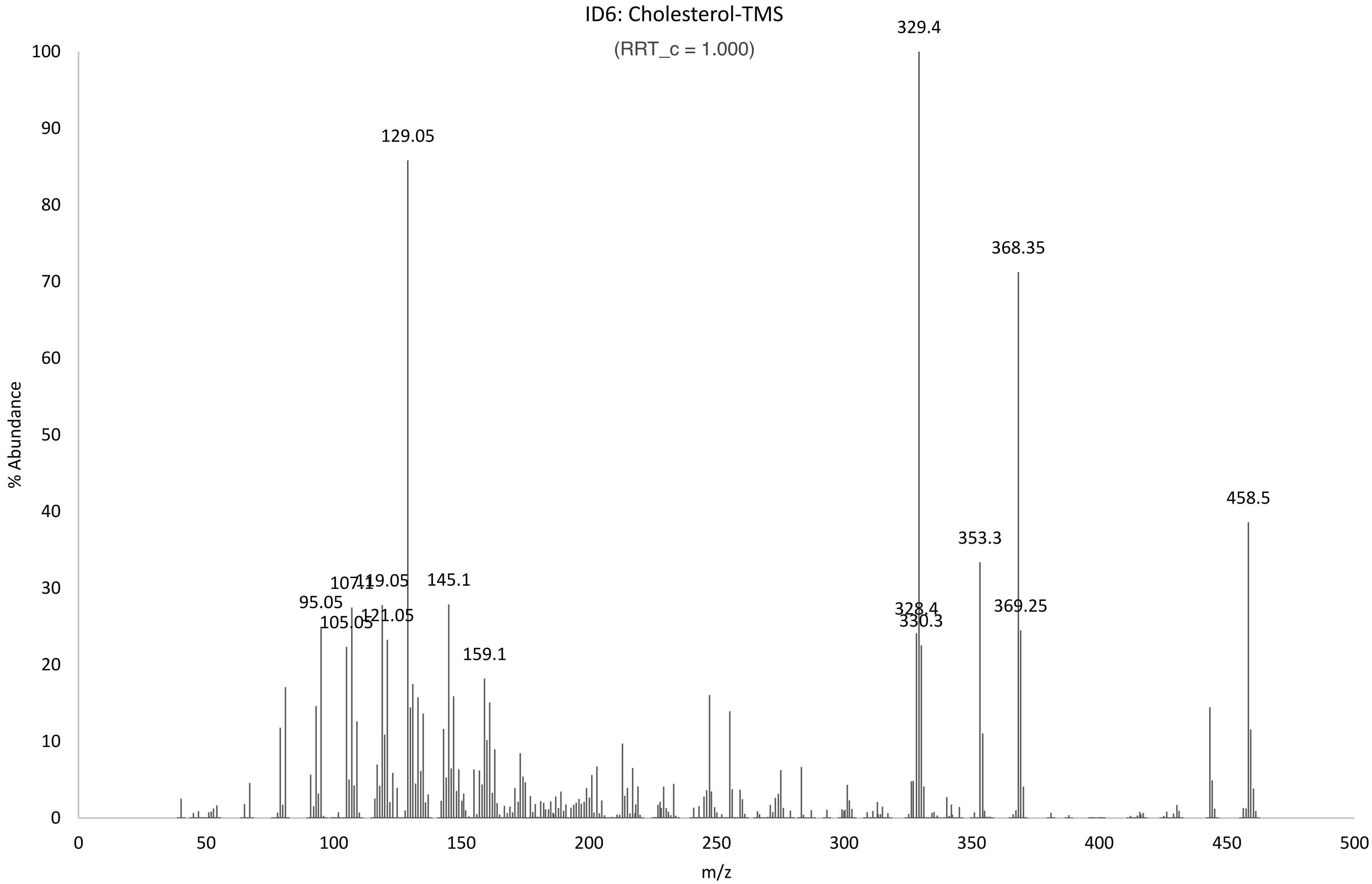
ID6: Cholesterol-TMS
(RRT_c = 1.000)
% Abundance
m/z
329.4
129.05
368.35
458.5
353.3
369.25
328.4
330.3
145.1
107.1
119.05
95.05
105.05
121.05
159.1
0
10
20
30
40
50
60
70
80
90
100
0
50
100
150
200
250
300
350
400
450
500

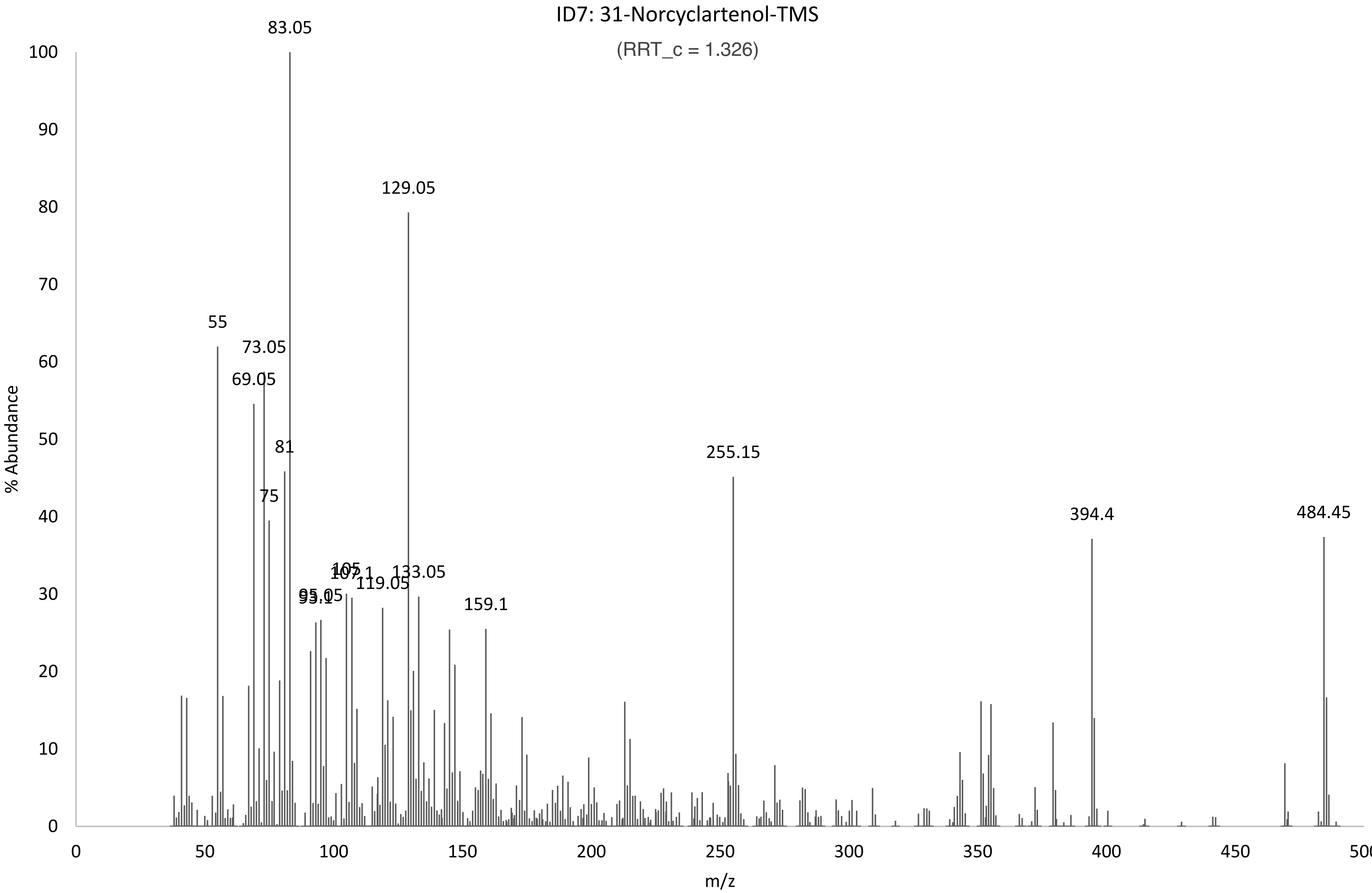
ID7: 31-Norcyclartenol-TMS
(RRT_c = 1.326)
% Abundance
m/z
0
10
20
30
40
50
60
70
80
90
100
0
50
100
150
200
250
300
350
400
450
500
83.05
129.05
55
73.05
69.05
81
75
105
107.1
133.05
119.05
95.05
93.1
159.1
255.15
394.4
484.45

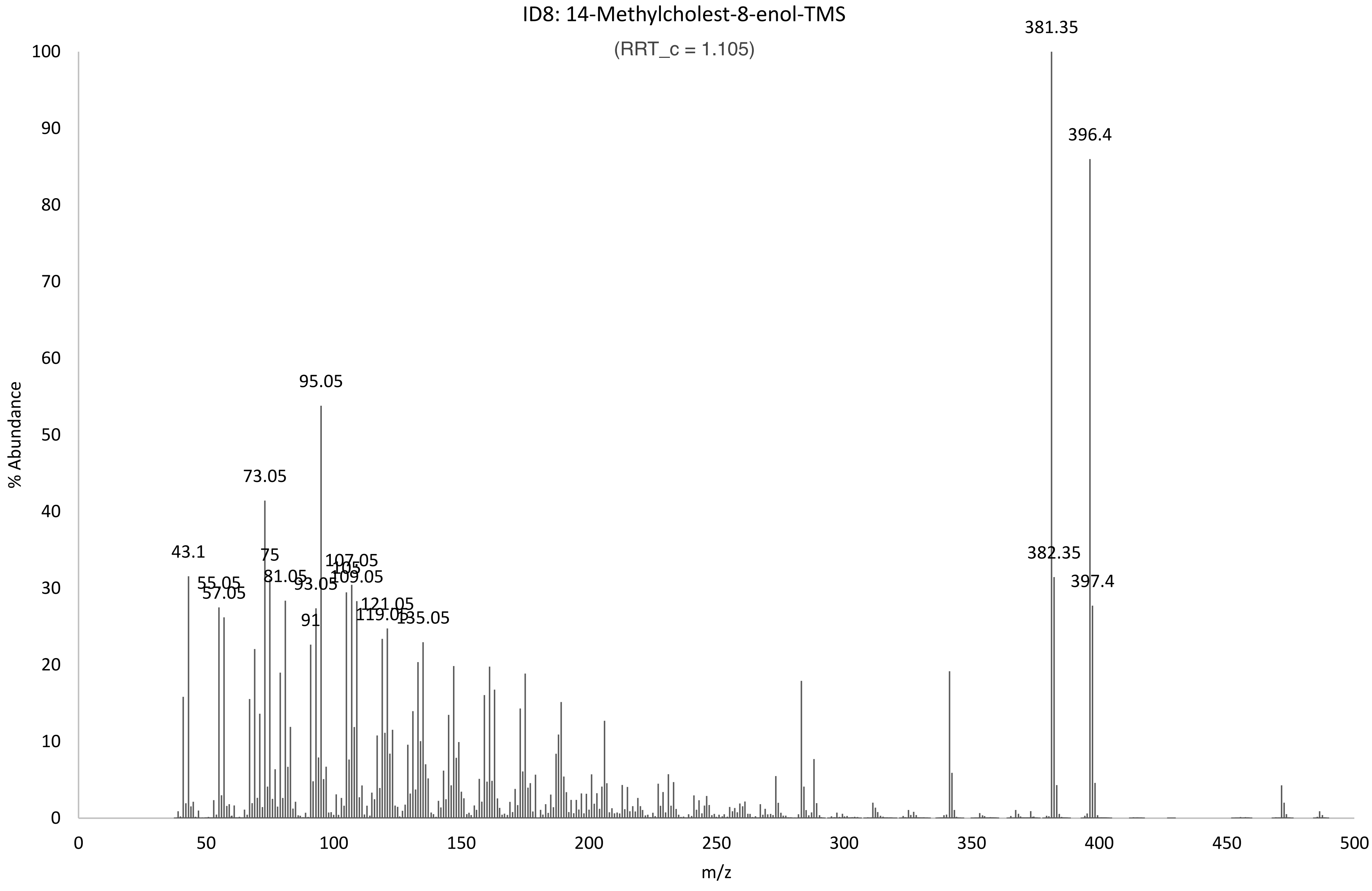

ID8: 14-Methylcholest-8-enol-TMS
(RRT_c = 1.105)
% Abundance
m/z
381.35
396.4
95.05
73.05
43.1
75
55.05
57.05
81.05
93.05
107.05
105
109.05
91
121.05
119.05
135.05
382.35
397.4
0
10
20
30
40
50
60
70
80
90
100
0
50
100
150
200
250
300
350
400
450
500

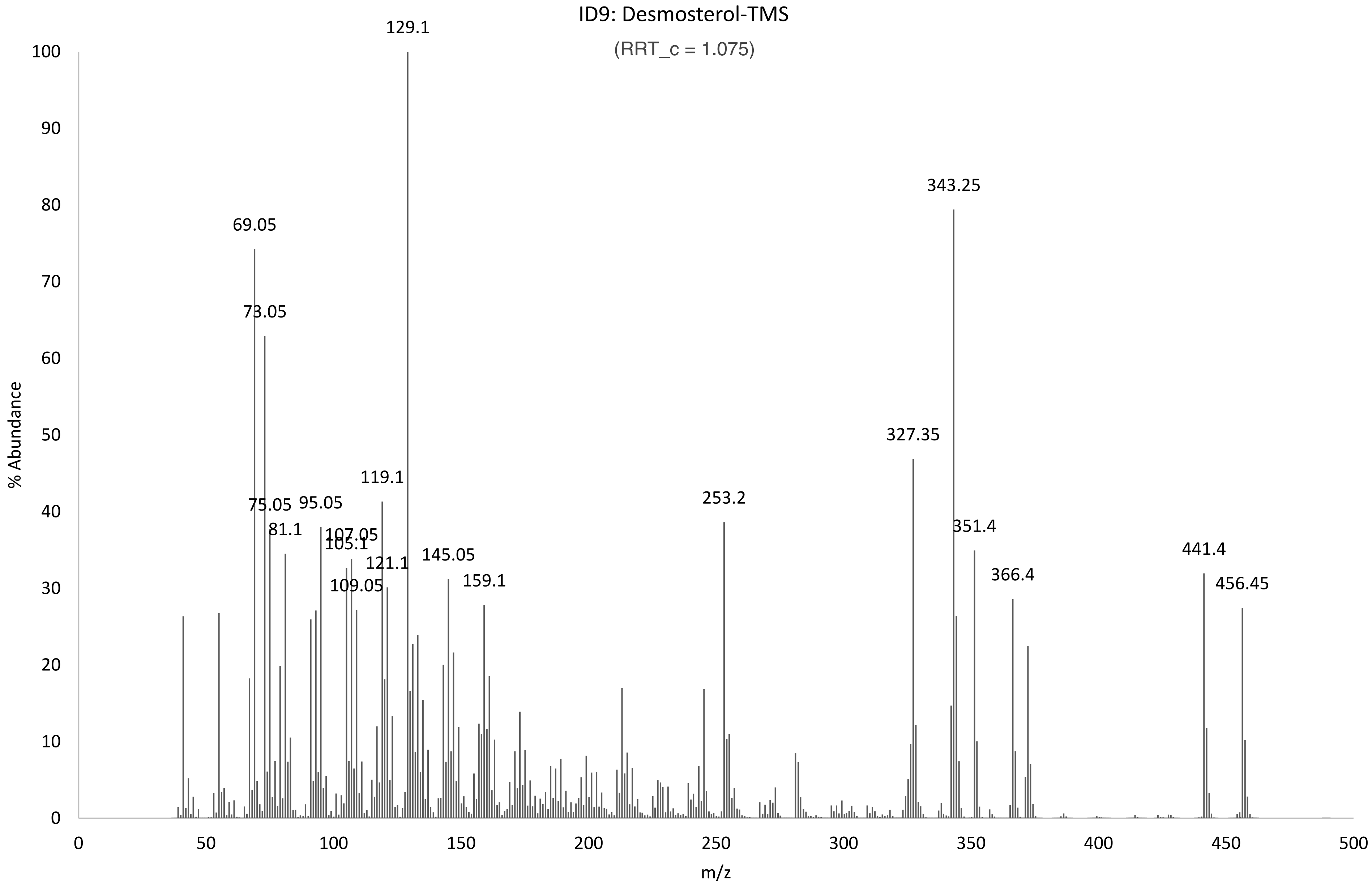
ID9: Desmosterol-TMS
(RRT_c = 1.075)
% Abundance
m/z
129.1
69.05
73.05
75.05
81.1
95.05
107.05
105.1
109.05
119.1
121.1
145.05
159.1
253.2
327.35
343.25
351.4
366.4
441.4
456.45
0
10
20
30
40
50
60
70
80
90
100
0
50
100
150
200
250
300
350
400
450
500

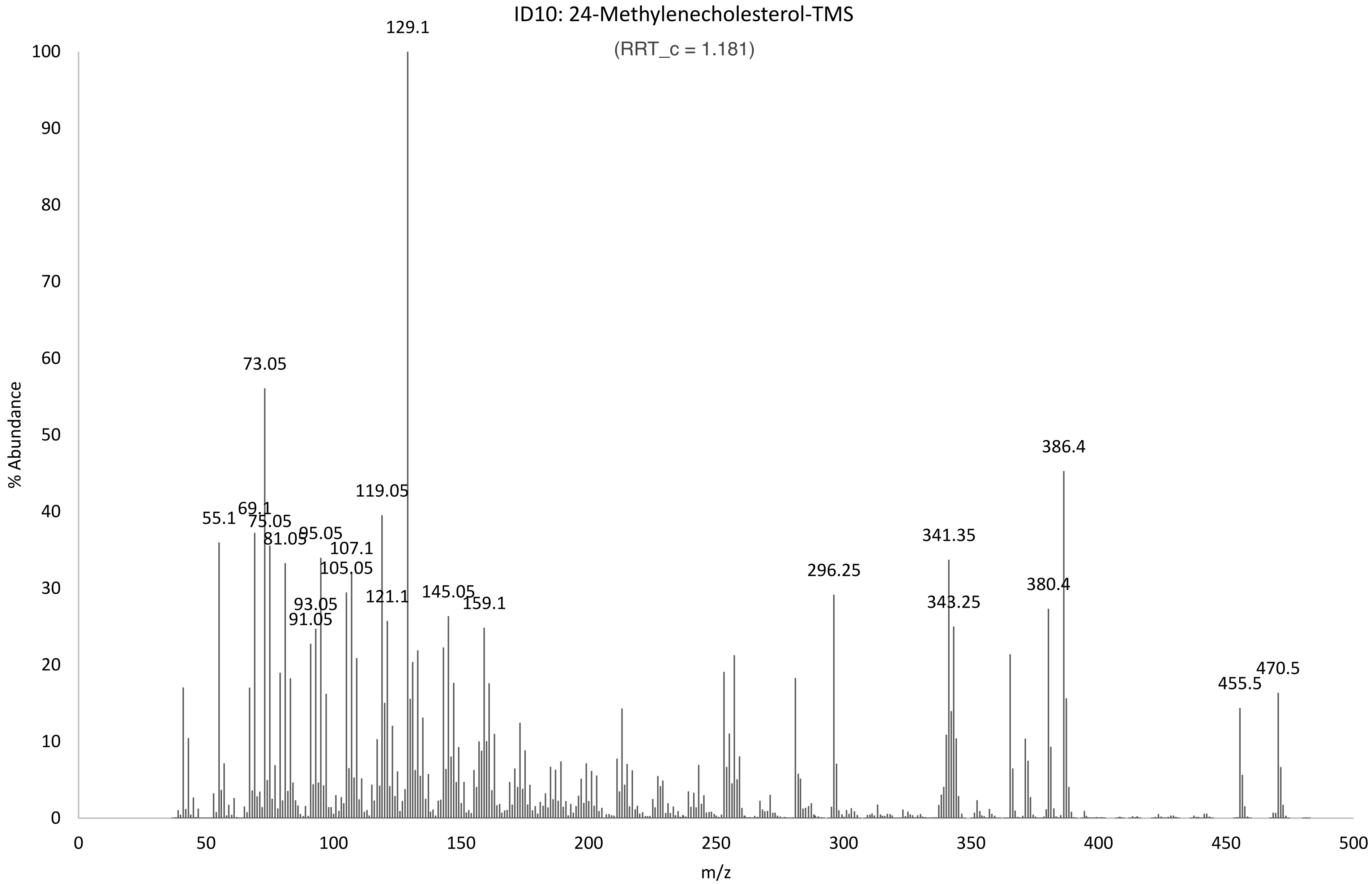

ID10: 24-Methylenecholesterol-TMS
(RRT_c = 1.181)
% Abundance
m/z
129.1
73.05
55.1
69.1
75.05
81.05
95.05
107.1
105.05
93.05
91.05
119.05
121.1
145.05
159.1
296.25
341.35
343.25
380.4
386.4
455.5
470.5
0
10
20
30
40
50
60
70
80
90
100
0
50
100
150
200
250
300
350
400
450
500

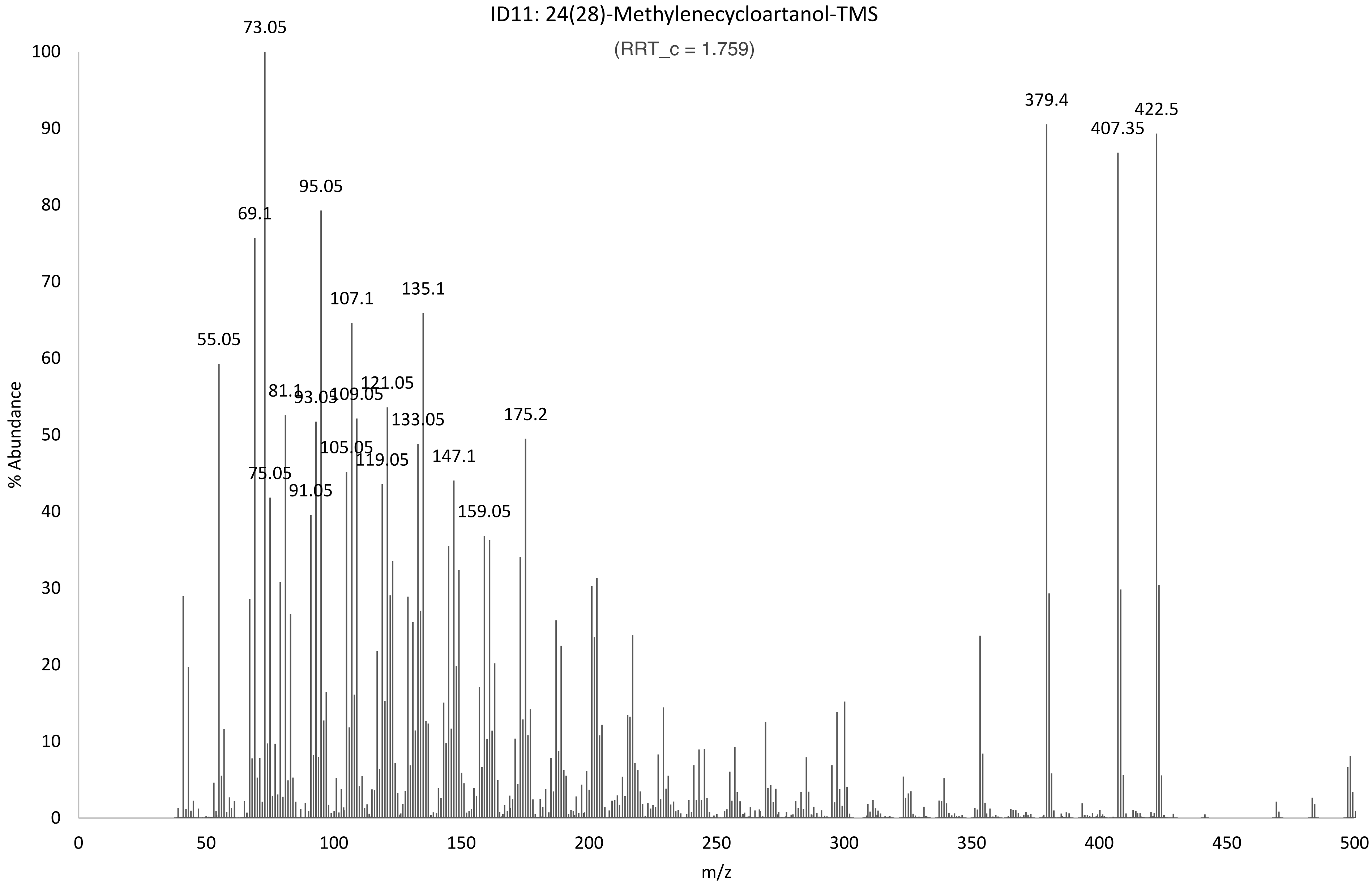
ID11: 24(28)-Methylenecycloartanol-TMS
(RRT_c = 1.759)
% Abundance
m/z
73.05
95.05
69.1
107.1
135.1
55.05
121.05
81.1
93.05
109.05
133.05
175.2
105.05
119.05
147.1
75.05
91.05
159.05
379.4
407.35
422.5
0
10
20
30
40
50
60
70
80
90
100
0
50
100
150
200
250
300
350
400
450
500

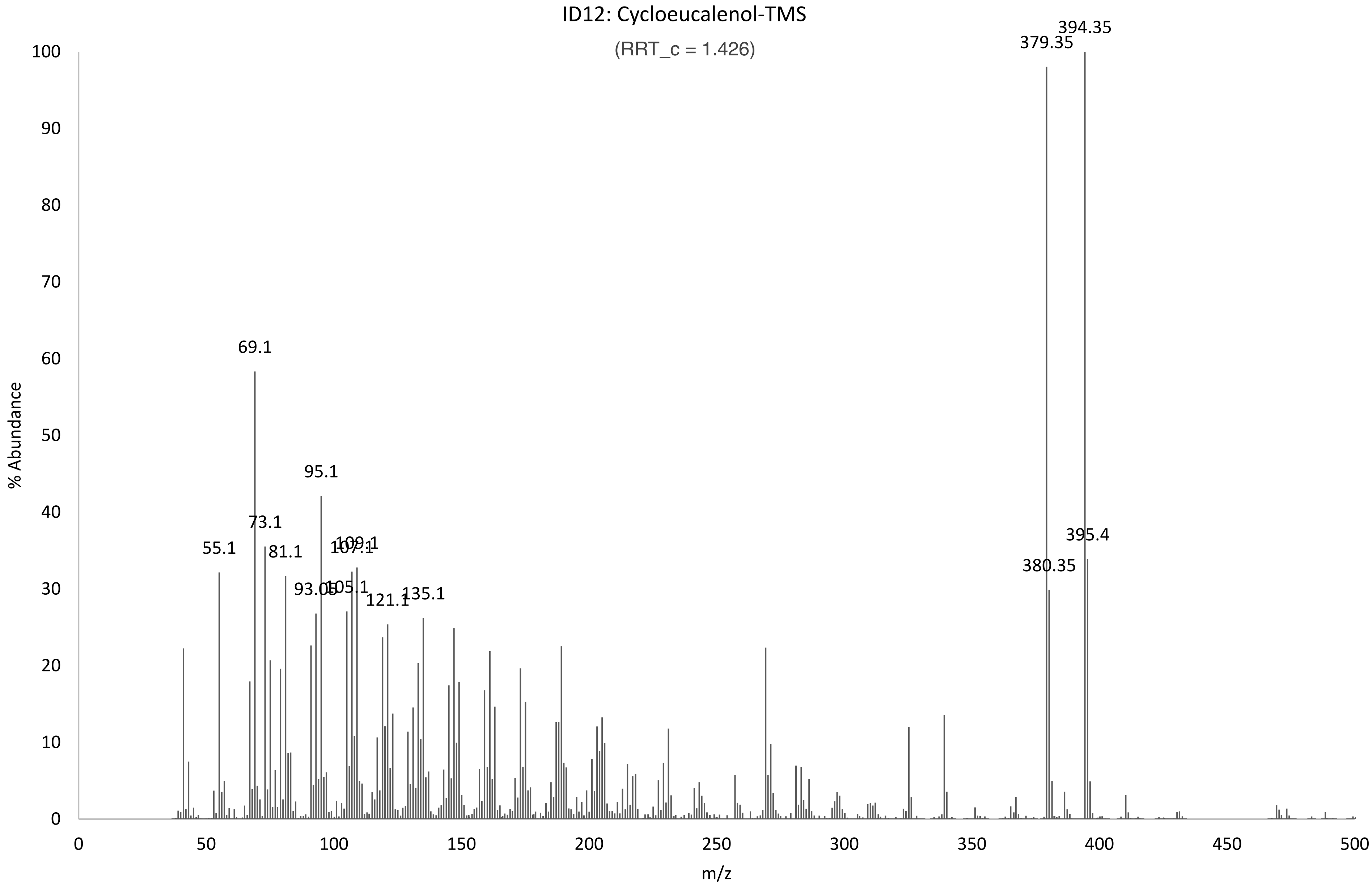
ID12: Cycloeucalenol-TMS
(RRT_c = 1.426)
% Abundance
m/z
69.1
95.1
73.1
55.1
81.1
109.1
107.1
93.05
105.1
121.1
135.1
379.35
394.35
395.4
380.35
0
10
20
30
40
50
60
70
80
90
100
0
50
100
150
200
250
300
350
400
450
500

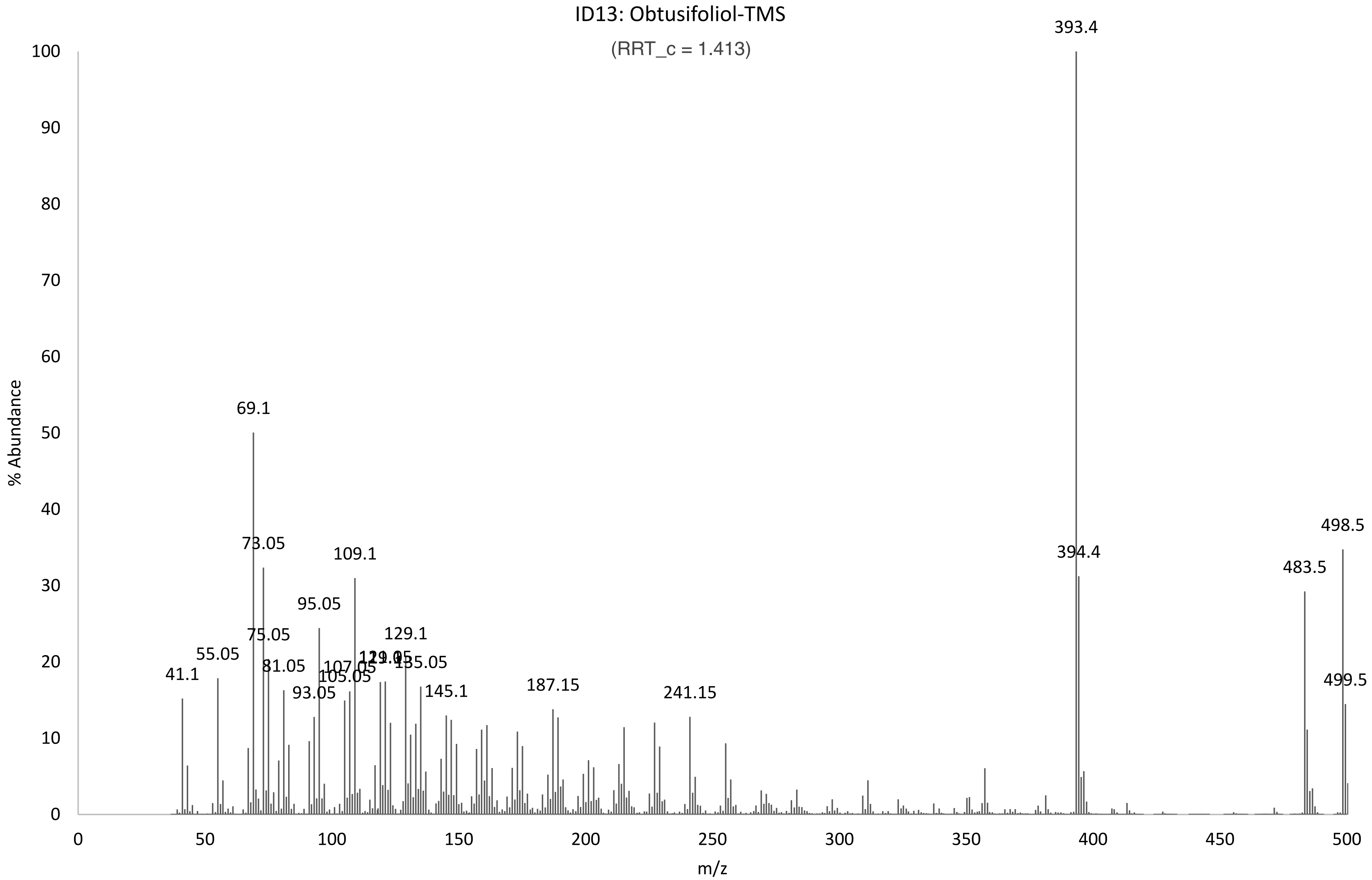
ID13: Obtusifoliol-TMS
(RRT_c = 1.413)
% Abundance
m/z
0
10
20
30
40
50
60
70
80
90
100
0
50
100
150
200
250
300
350
400
450
500
393.4
69.1
73.05
109.1
95.05
75.05
55.05
41.1
81.05
93.05
105.05
107.05
129.1
135.05
145.1
187.15
241.15
394.4
483.5
498.5
499.5

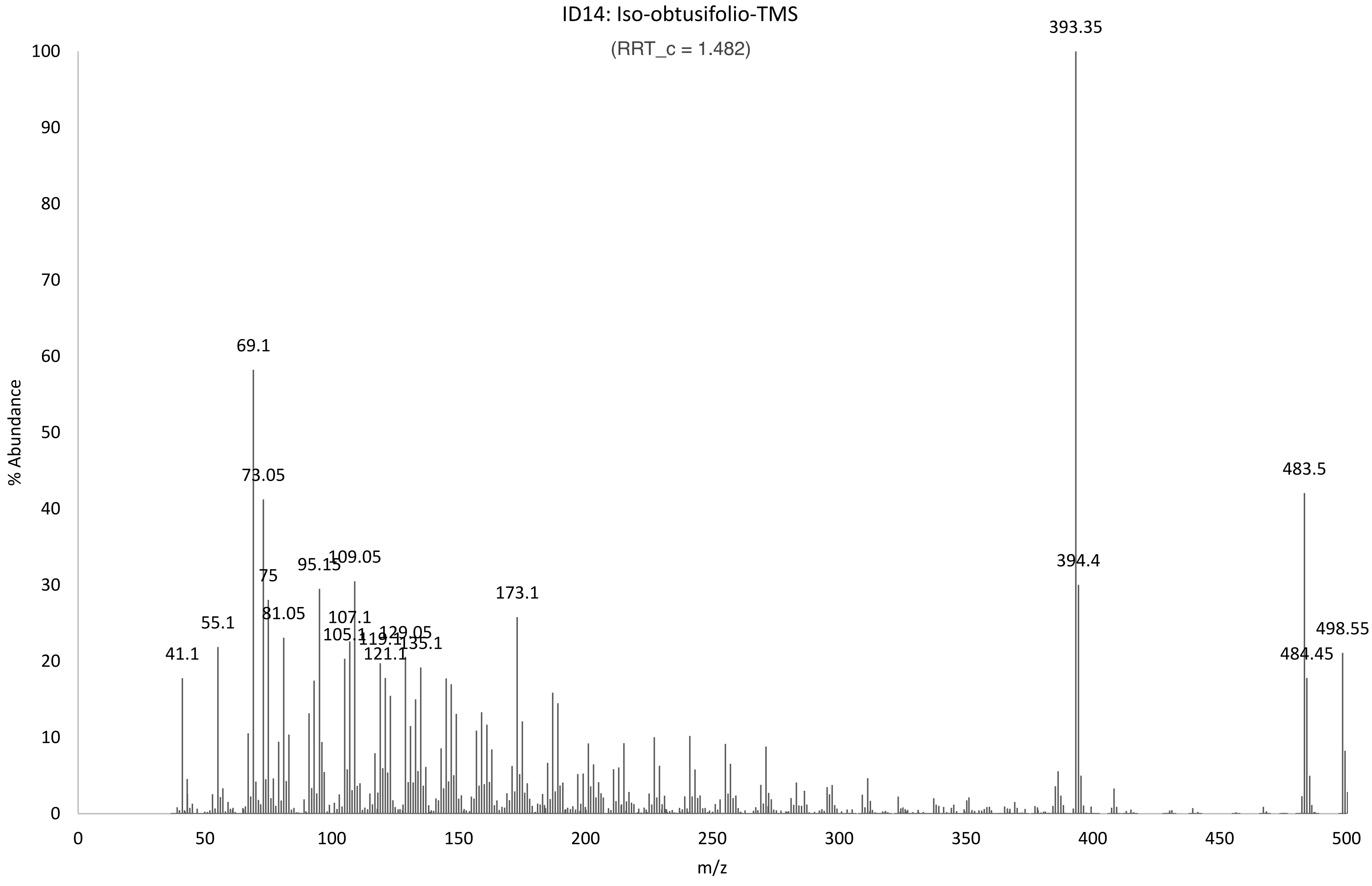
ID14: Iso-obtusifolio-TMS
(RRT_c = 1.482)
% Abundance
m/z
0
10
20
30
40
50
60
70
80
90
100
0
50
100
150
200
250
300
350
400
450
500
393.35
69.1
483.5
73.05
109.05
95.15
394.4
75
173.1
81.05
107.1
55.1
105.1
119.1
129.05
135.1
121.1
41.1
498.55
484.45

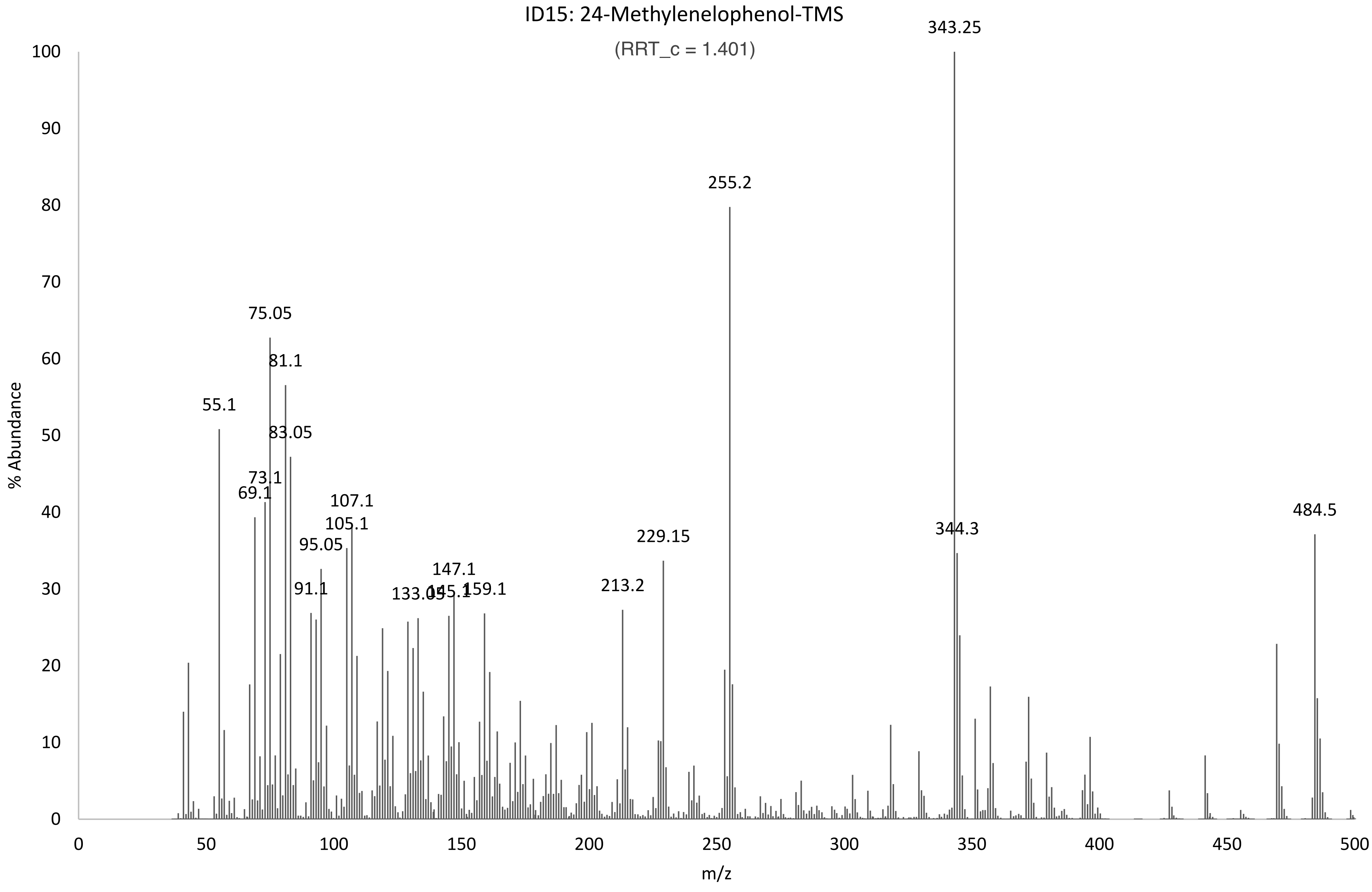
ID15: 24-Methylenelophenol-TMS
(RRT_c = 1.401)
% Abundance
m/z
343.25
255.2
75.05
81.1
55.1
83.05
73.1
69.1
107.1
105.1
95.05
91.1
133.05
145.1
147.1
159.1
213.2
229.15
344.3
484.5
0
10
20
30
40
50
60
70
80
90
100
0
50
100
150
200
250
300
350
400
450
500

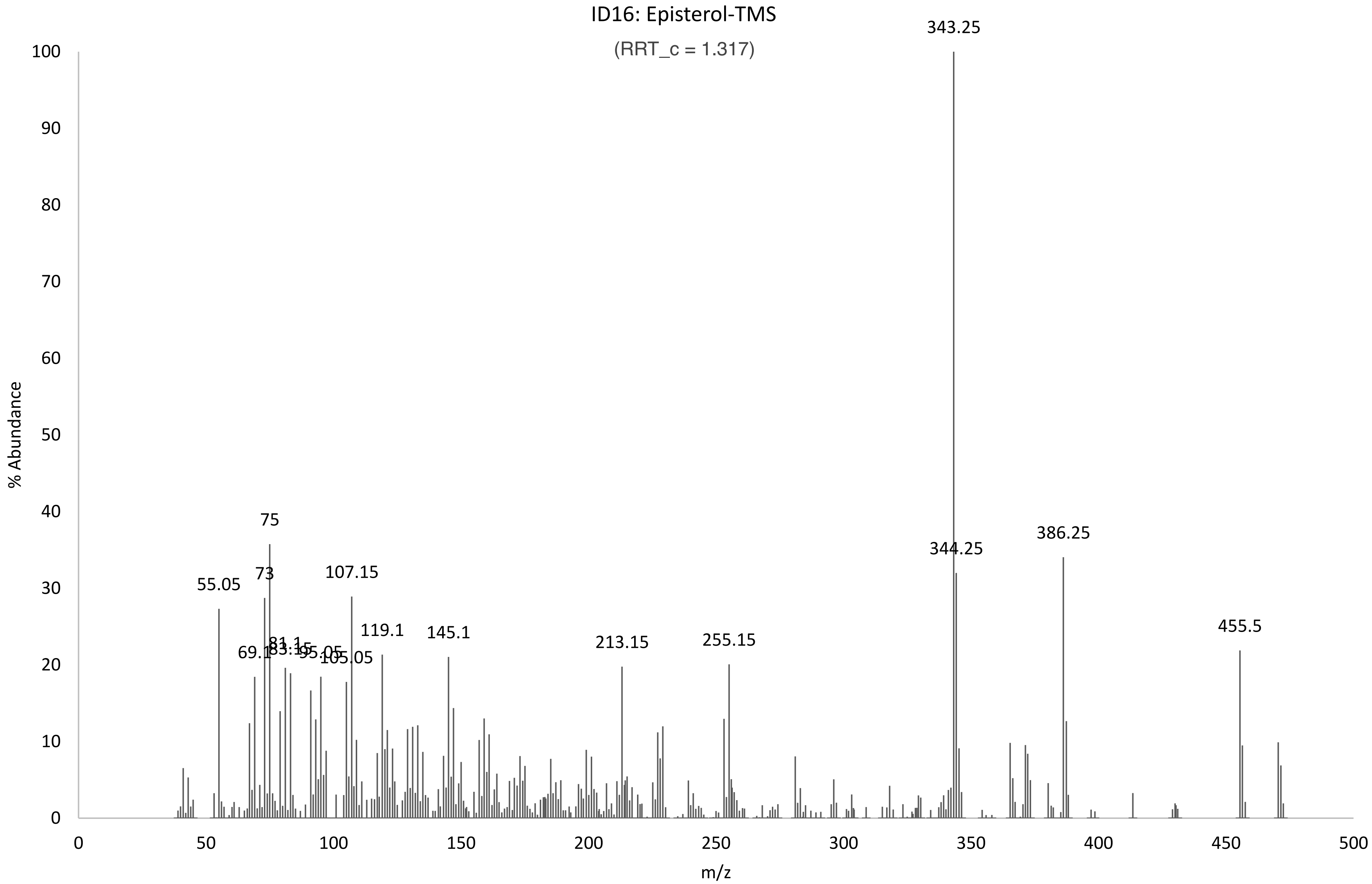
ID16: Episterol-TMS
(RRT_c = 1.317)
343.25
344.25
386.25
455.5
75
73
55.05
107.15
119.1
145.1
213.15
255.15
69.1
81.1
83.15
95.05
105.05
% Abundance
m/z
0
10
20
30
40
50
60
70
80
90
100
0
50
100
150
200
250
300
350
400
450
500

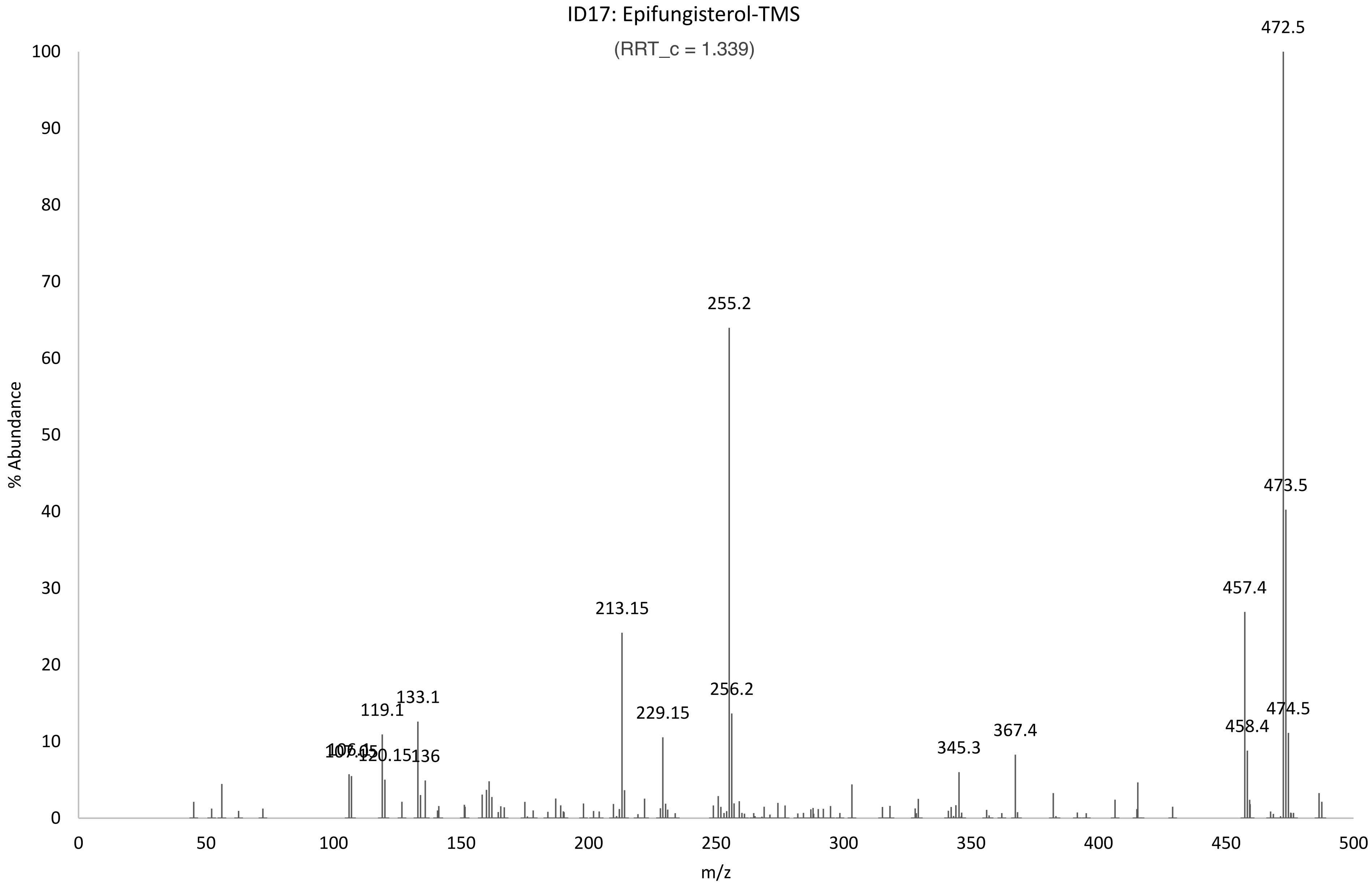
ID17: Epifungisterol-TMS
(RRT_c = 1.339)
% Abundance
m/z
0
10
20
30
40
50
60
70
80
90
100
0
50
100
150
200
250
300
350
400
450
500
472.5
255.2
473.5
457.4
213.15
256.2
133.1
119.1
229.15
474.5
458.4
367.4
345.3
136
120.15
106.15
107.15

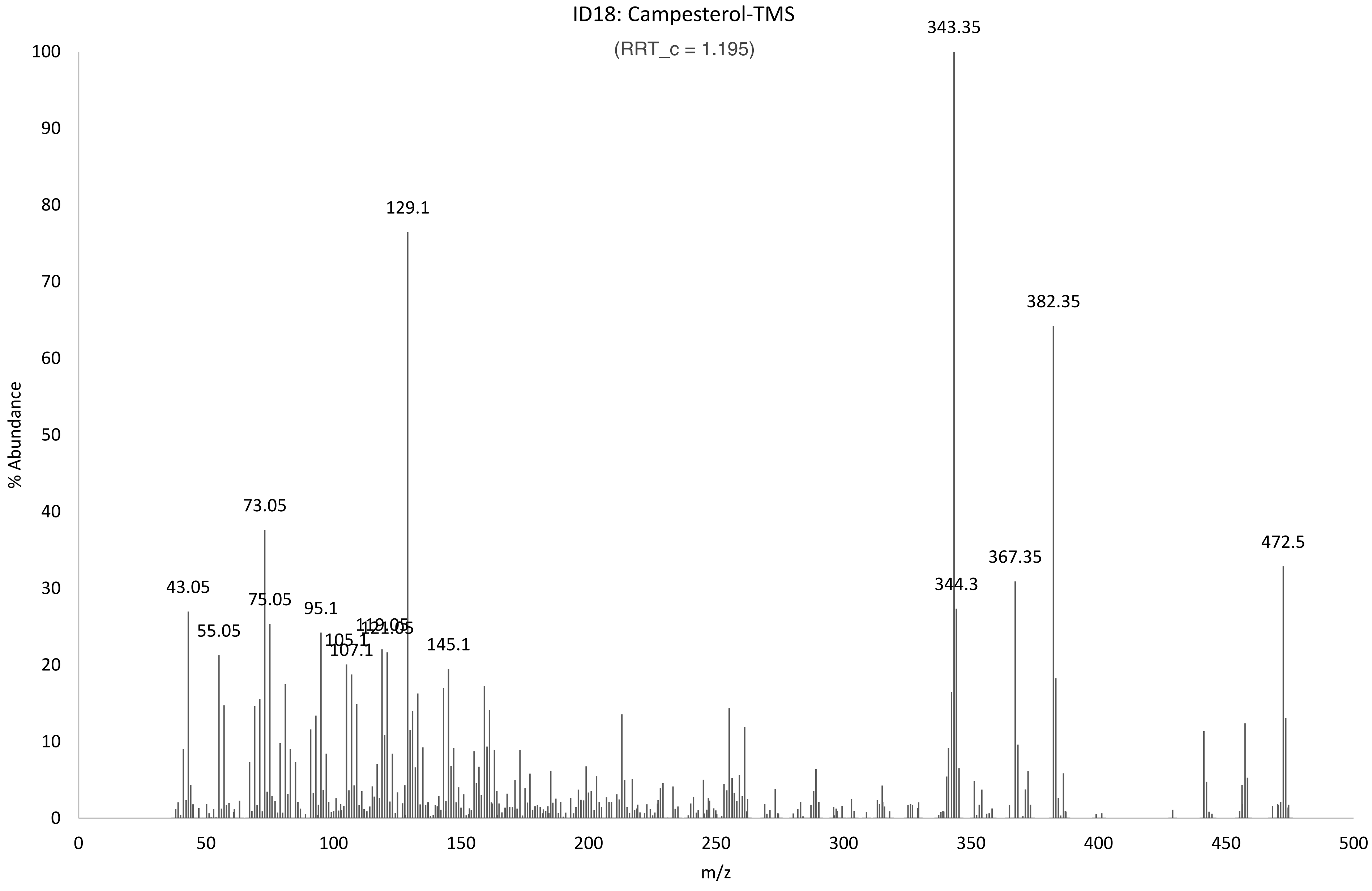

ID18: Campesterol-TMS
(RRT_c = 1.195)
% Abundance
m/z
100
90
80
70
60
50
40
30
20
10
0
0
50
100
150
200
250
300
350
400
450
500
343.35
129.1
382.35
73.05
472.5
43.05
367.35
344.3
75.05
95.1
55.05
119.05
121.05
105.1
107.1
145.1

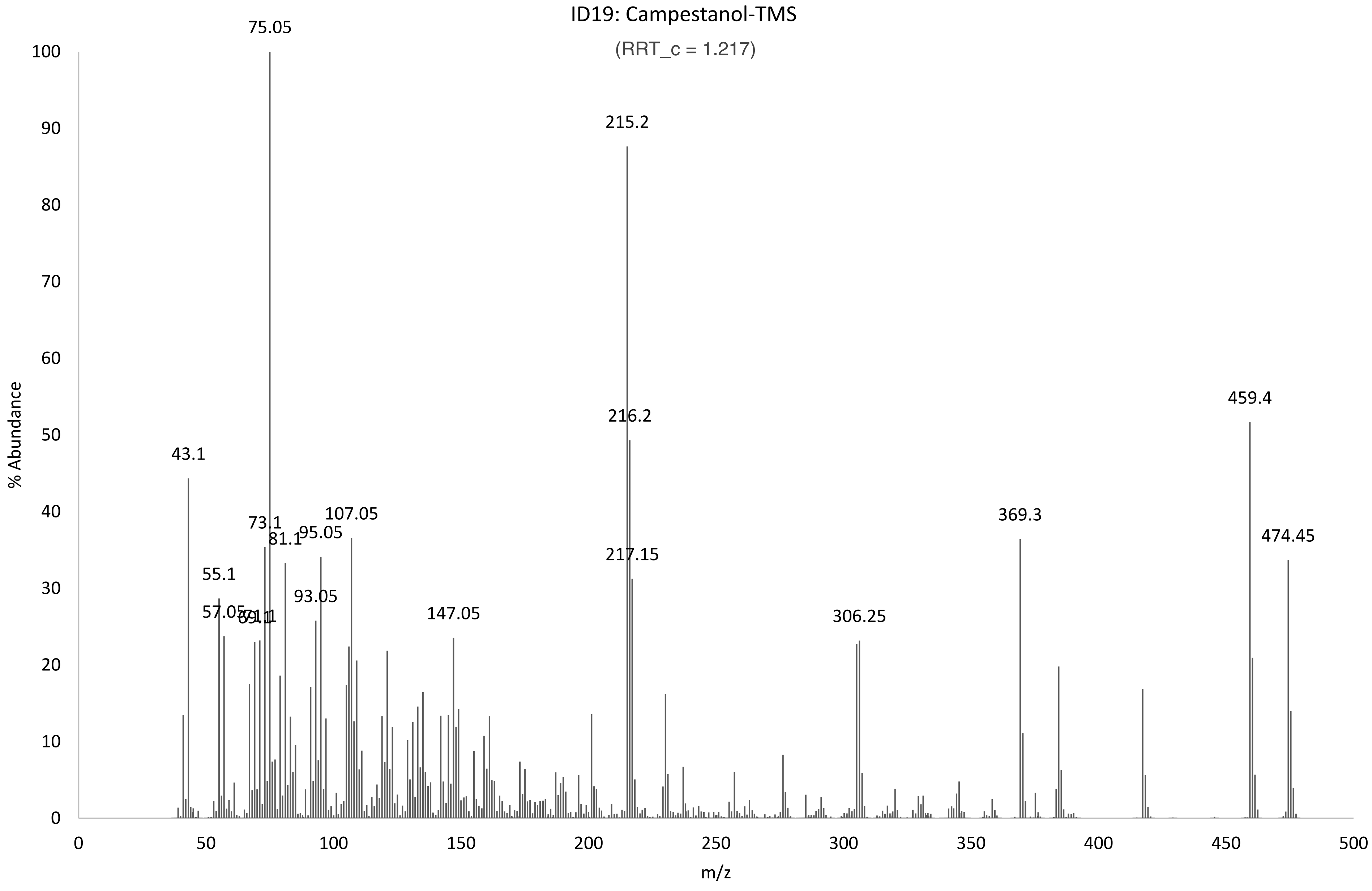
ID19: Campestanol-TMS
(RRT_c = 1.217)
% Abundance
m/z
75.05
215.2
216.2
217.15
43.1
55.1
57.05
69.1
71.1
73.1
81.1
93.05
95.05
107.05
147.05
306.25
369.3
459.4
474.45
0
10
20
30
40
50
60
70
80
90
100
0
50
100
150
200
250
300
350
400
450
500

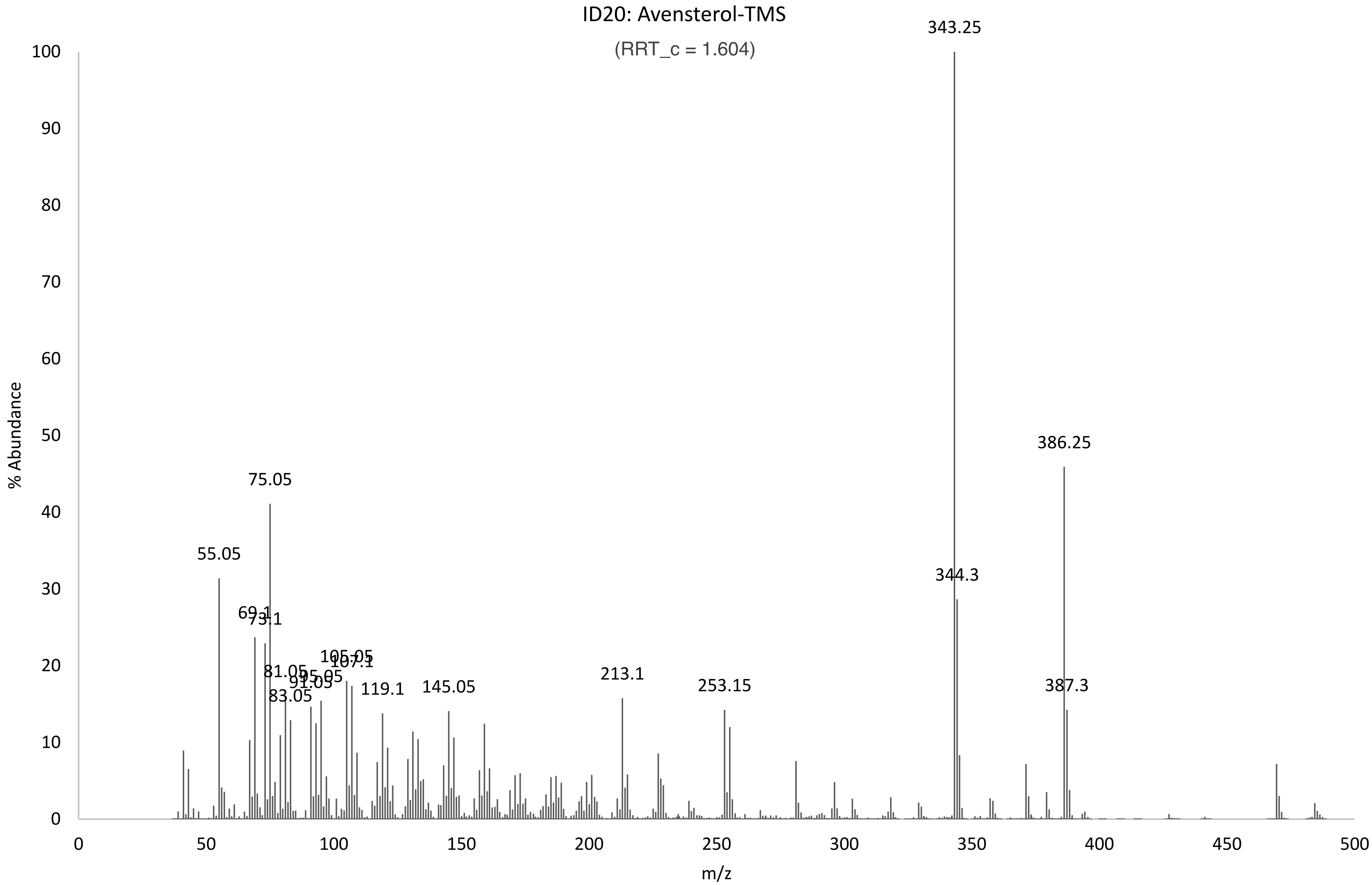
ID20: Avensterol-TMS
(RRT_c = 1.604)
% Abundance
m/z
0
10
20
30
40
50
60
70
80
90
100
0
50
100
150
200
250
300
350
400
450
500
343.25
386.25
75.05
55.05
344.3
69.1
73.1
105.05
107.1
81.05
95.05
91.05
83.05
119.1
145.05
213.1
253.15
387.3

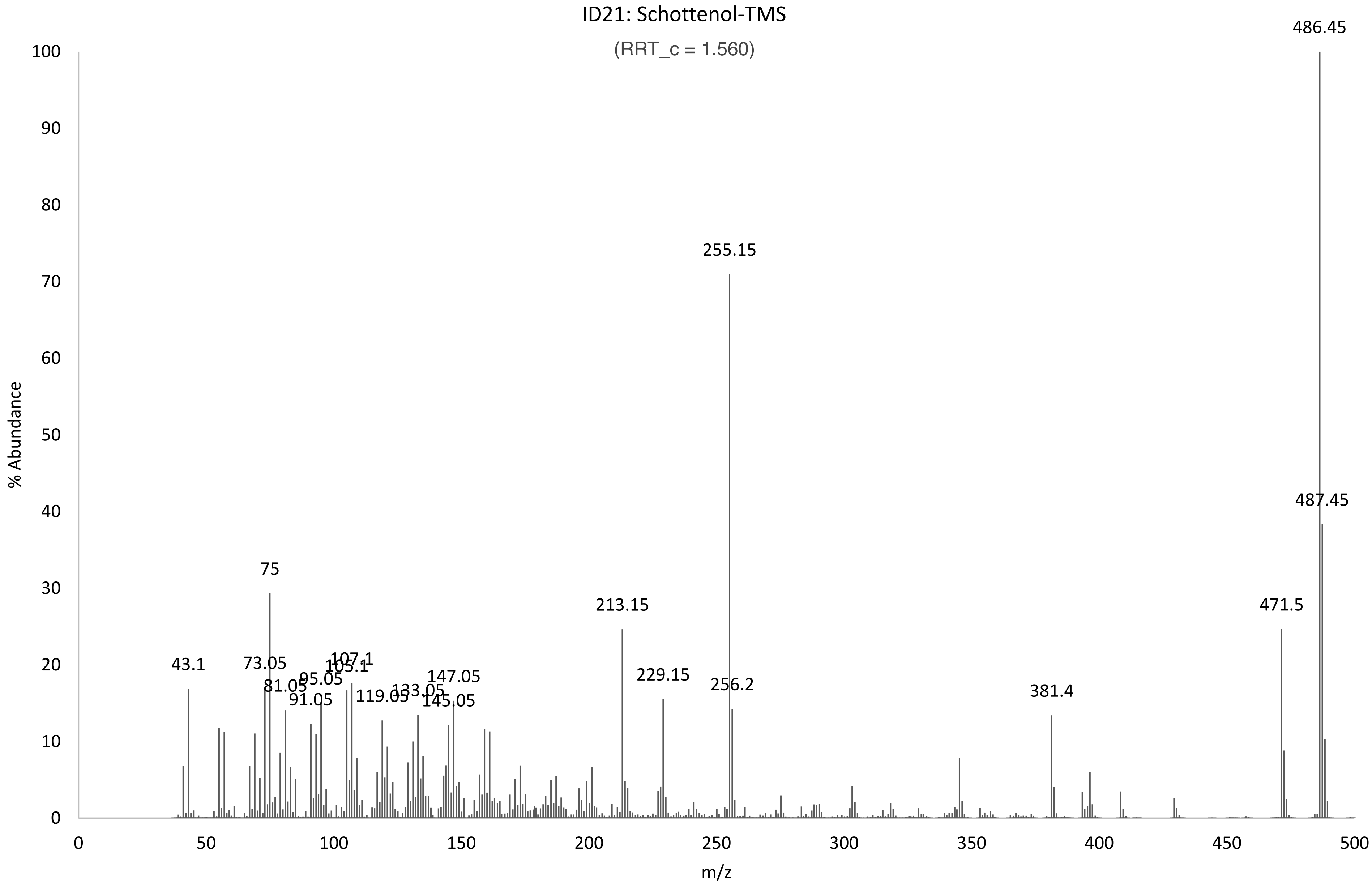
ID21: Schottenol-TMS
(RRT_c = 1.560)
% Abundance
m/z
0
10
20
30
40
50
60
70
80
90
100
0
50
100
150
200
250
300
350
400
450
500
486.45
487.45
471.5
255.15
256.2
381.4
213.15
229.15
75
73.05
43.1
81.05
91.05
95.05
105.1
107.1
119.05
133.05
145.05
147.05

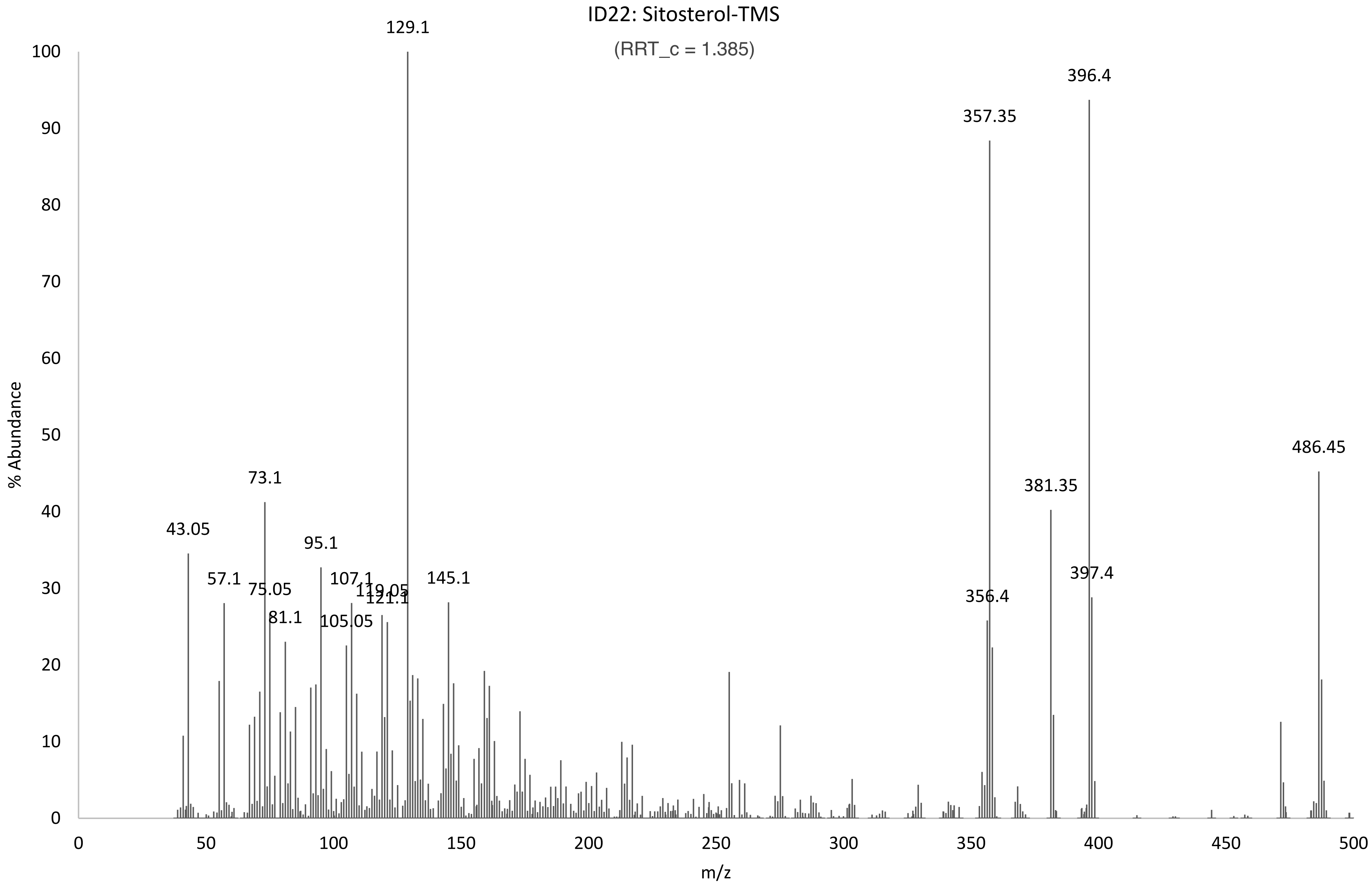
ID22: Sitosterol-TMS
(RRT_c = 1.385)
% Abundance
m/z
129.1
396.4
357.35
486.45
381.35
397.4
356.4
73.1
43.05
95.1
57.1
107.1
75.05
119.05
121.1
145.1
81.1
105.05
0
10
20
30
40
50
60
70
80
90
100
0
50
100
150
200
250
300
350
400
450
500

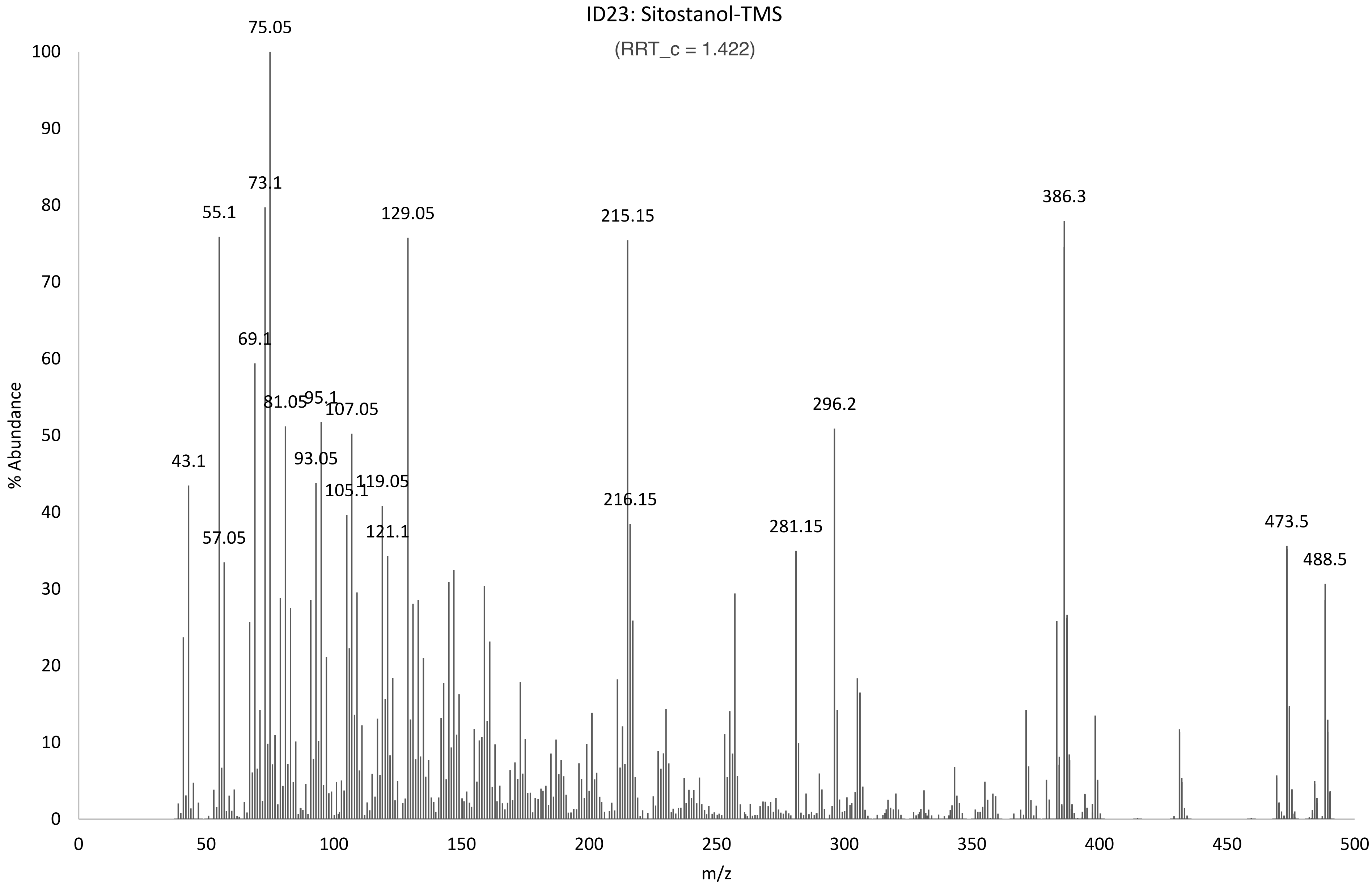
ID23: Sitostanol-TMS
(RRT_c = 1.422)
% Abundance
m/z
75.05
73.1
55.1
129.05
215.15
386.3
69.1
81.05
95.1
107.05
296.2
43.1
93.05
119.05
105.1
216.15
57.05
121.1
281.15
473.5
488.5
0
10
20
30
40
50
60
70
80
90
100
0
50
100
150
200
250
300
350
400
450
500

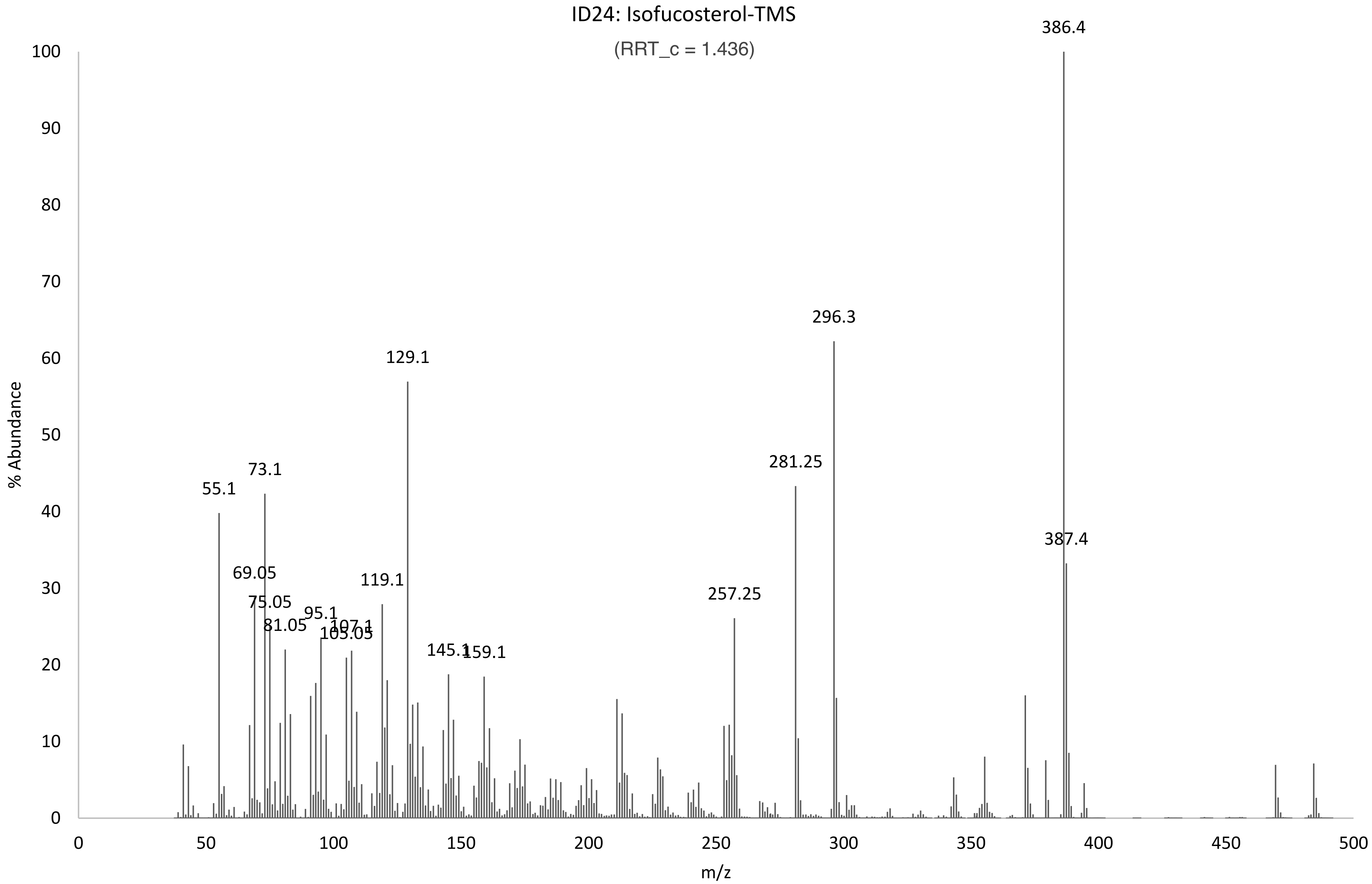
ID24: Isofucosterol-TMS
(RRT_c = 1.436)
% Abundance
m/z
386.4
296.3
129.1
281.25
73.1
55.1
387.4
69.05
119.1
257.25
75.05
95.1
81.05
107.1
105.05
145.1
159.1
0
10
20
30
40
50
60
70
80
90
100
0
50
100
150
200
250
300
350
400
450
500

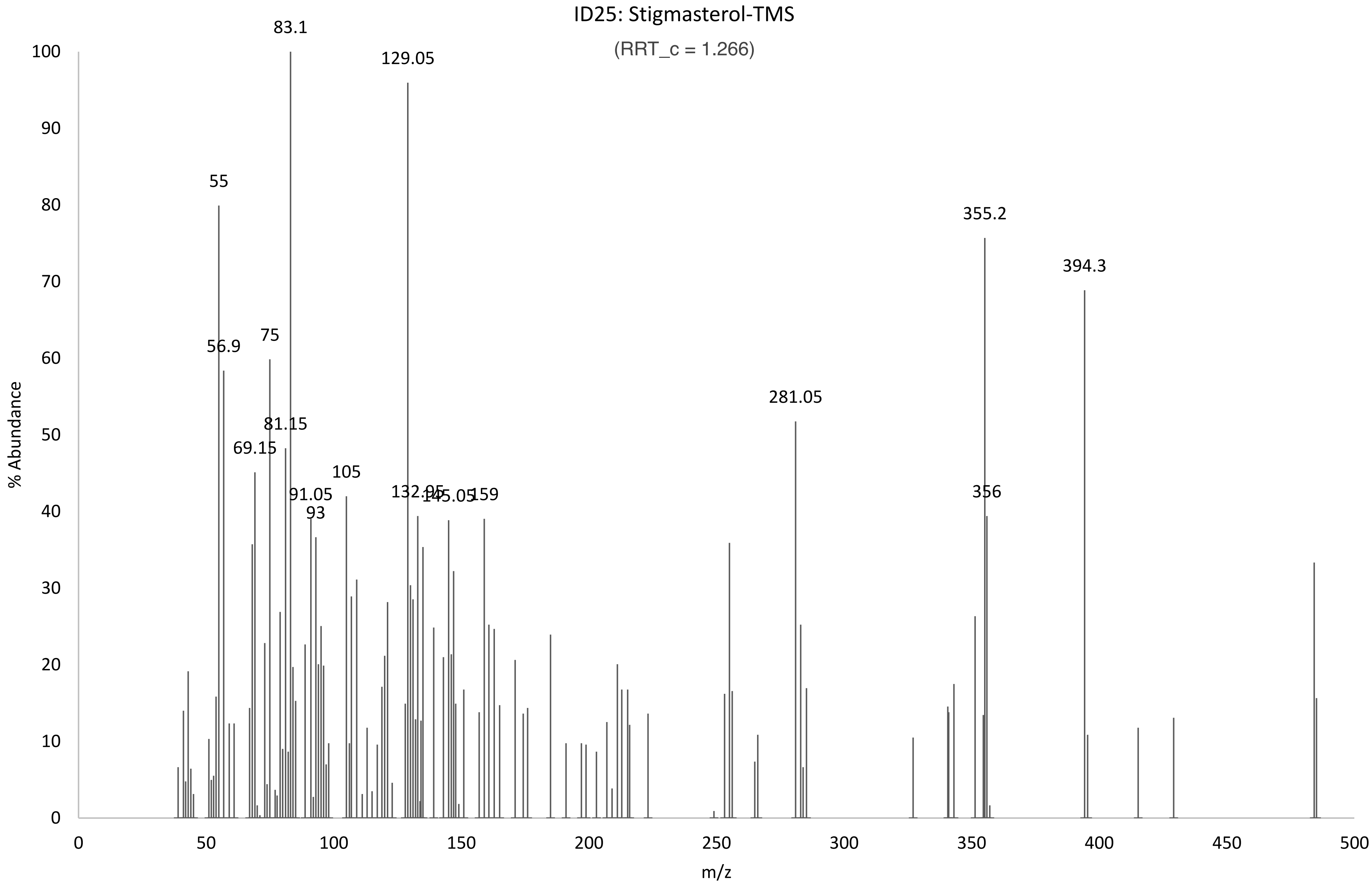
ID25: Stigmasterol-TMS
(RRT_c = 1.266)
% Abundance
m/z
83.1
129.05
55
355.2
394.3
56.9
75
281.05
81.15
69.15
105
91.05
93
132.05
145.05
159
356
0
10
20
30
40
50
60
70
80
90
100
0
50
100
150
200
250
300
350
400
450
500

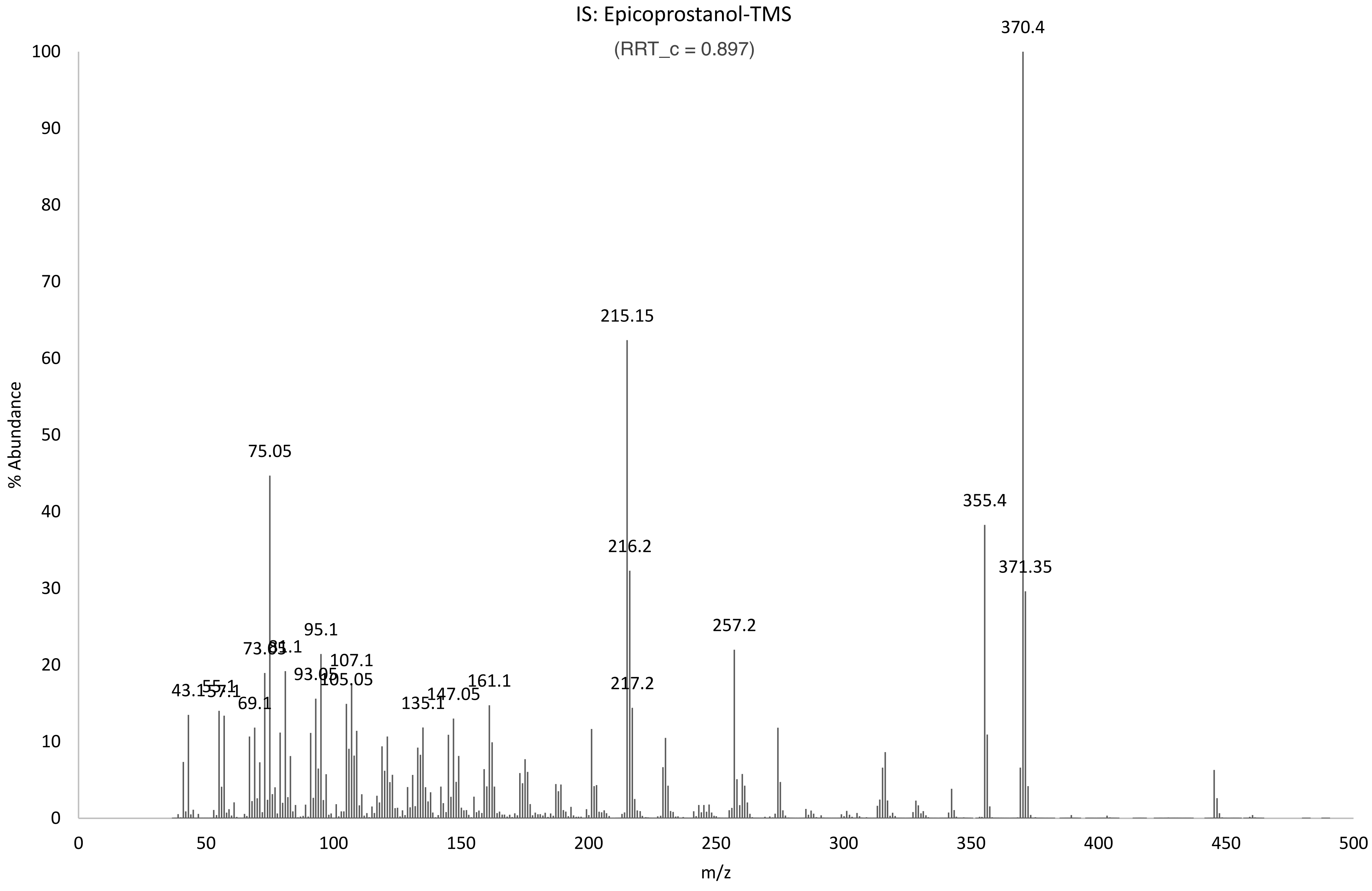
IS: Epicoprostanol-TMS
(RRT_c = 0.897)
% Abundance
m/z
370.4
215.15
75.05
355.4
216.2
371.35
95.1
257.2
73.05
81.1
107.1
93.05
105.05
161.1
217.2
43.1
55.1
57.1
147.05
69.1
135.1
